# Deciphering novel mitochondrial signatures: multi-omics analysis uncovers cross-disease markers and oligodendrocyte pathways in Alzheimer’s disease and glioblastoma

**DOI:** 10.3389/fnagi.2025.1536142

**Published:** 2025-02-13

**Authors:** Xuan Xu, Jiaqi Wang, Tong Chen, Shuaibin Wang, Fei Wang, Junwen He, Xiang-Yu Meng, Yin Shen

**Affiliations:** ^1^School of Life Sciences, Anhui Medical University, Hefei, Anhui, China; ^2^School of Biomedical Engineering, Anhui Medical University, Hefei, Anhui, China; ^3^School of Basic Medical Science, Anhui Medical University, Hefei, Anhui, China; ^4^College of Informatics, Huazhong Agricultural University, Hubei, Wuhan, China; ^5^School of Basic Medical Sciences, Medical School, Hubei Minzu University, Enshi, Hubei, China

**Keywords:** Alzheimer’s disease, glioblastoma, mitochondria, multi-omics, biomarker

## Abstract

**Introduction:**

Alzheimer’s disease (AD) and glioblastoma (GBM) are severe neurological disorders that pose significant global healthcare challenges. Despite extensive research, the molecular mechanisms, particularly those involving mitochondrial dysfunction, remain poorly understood. A major limitation in current studies is the lack of cell-specific markers that effectively represent mitochondrial dynamics in AD and GBM.

**Methods:**

In this study, we analyzed single-cell transcriptomic data using 10 machine learning algorithms to identify mitochondria-associated cell-specific markers. We validated these markers through the integration of gene expression and methylation data across diverse cell types. Our dataset comprised single-nucleus RNA sequencing (snRNA-seq) from AD patients, single-cell RNA sequencing (scRNA-seq) from GBM patients, and additional DNA methylation and transcriptomic data from the ROSMAP, ADNI, TCGA, and CGGA cohorts.

**Results:**

Our analysis identified four significant cross-disease mitochondrial markers: *EFHD1, SASH1, FAM110B,* and *SLC25A18*. These markers showed both shared and unique expression profiles in AD and GBM, suggesting a common mitochondrial mechanism contributing to both diseases. Additionally, oligodendrocytes and their interactions with astrocytes were implicated in disease progression, particularly through the APP signaling pathway. Key hub genes, such as *HS6ST3* and *TUBB2B*, were identified across different cellular subpopulations, highlighting a cell-specific co-expression network linked to mitochondrial function.

## 1 Introduction

Alzheimer’s disease (AD) and glioblastoma (GBM) are major neurological disorders that impact the brain’s cellular networks in distinct but complex ways. AD, the most prevalent form of dementia, is characterized by progressive cognitive decline, while GBM is an aggressive brain tumor noted for rapid growth and resistance to conventional therapies (Scheltens et al., 2021; Price et al., 2024). Interestingly, there is a significant overlap between these conditions, particularly among older adults. Approximately 42% of GBM patients aged 60–82 display AD-related pathology, suggesting potential shared pathological mechanisms (Xia et al., 2023). Epidemiological evidence indicates an inverse correlation between AD and GBM, but their co-occurrence can substantially affect disease progression and treatment outcomes (Lanni et al., 2021). Animal models have further demonstrated that the presence of AD pathology influences GBM tumor growth, disease progression, and immune responses (Broekman et al., 2018). This supports the hypothesis that AD and GBM may share underlying etiological factors, possibly linked to protein misfolding or biochemical changes characteristic of dementia (Lehrer, 2010; Sánchez-Valle et al., 2017). These findings highlight the importance of assessing cognitive status and AD pathology in elderly GBM patients, as it may inform treatment strategies, particularly in the context of radiotherapy (Shi and Huang, 2023; Fetcko-Fayad et al., 2024). Further investigation is warranted to uncover the underlying mechanisms and shared pathways between these two devastating neurological conditions.

Mitochondrial dysfunction is a critical feature of both AD and GBM. In AD, mitochondrial impairment is a hallmark that drives neuronal death and cognitive decline (Calvo-Rodriguez and Bacskai, 2021). Mitochondrial dysfunction in AD leads to reduced energy production, increased oxidative stress, and compromised mitochondrial dynamics, thereby impairing cellular quality control and exacerbating neuronal damage (Ebanks et al., 2020). These findings suggest that strategies to improve mitochondrial function and mitigate oxidative stress may benefit AD patients. Conversely, in GBM, mitochondria play a key role in supporting the high metabolic demands of rapidly proliferating tumor cells (Thakur et al., 2022). GBM cells reprogram their metabolism to enhance mitochondrial respiration and can acquire mitochondria from neighboring healthy cells, thereby boosting their tumor-forming potential (Zhang et al., 2023b). This metabolic reprogramming and mitochondrial hijacking represent promising therapeutic targets, with emerging drugs that inhibit mitochondrial apoptosis and mitophagy showing potential efficacy in GBM treatment. Despite considerable research, a comprehensive understanding of the mitochondrial mechanisms underpinning AD and GBM remains elusive. The advent of single-cell RNA sequencing (scRNA-seq) has opened new avenues for investigating cellular heterogeneity, offering unprecedented insight into disease progression at a cellular level (Lin P. et al., 2024). However, the full potential of scRNA-seq to elucidate mitochondrial functions in AD and GBM has yet to be realized (Wang et al., 2022).

This study aims to address this gap by utilizing scRNA-seq data combined with machine learning algorithms to identify mitochondrial-related biomarkers with differential expression across diverse cell types in AD and GBM. By integrating scRNA-seq with epigenetic and transcriptomic analyses, we aim to elucidate the regulatory mechanisms governing mitochondrial gene expression, thereby providing a more comprehensive view of their roles across various cellular contexts. The application of machine learning to multi-omics data offers a powerful framework to dissect the intricate relationships between mitochondrial function and cellular identity (Baysoy et al., 2023). This dual-pronged approach is expected to reveal novel biomarkers and regulatory pathways that traditional bulk tissue analyses may have overlooked (Zhu et al., 2020). By identifying cell type-specific mitochondrial signatures, our research seeks to refine our understanding of AD and GBM, potentially uncovering new therapeutic targets.

**Figure 1:**
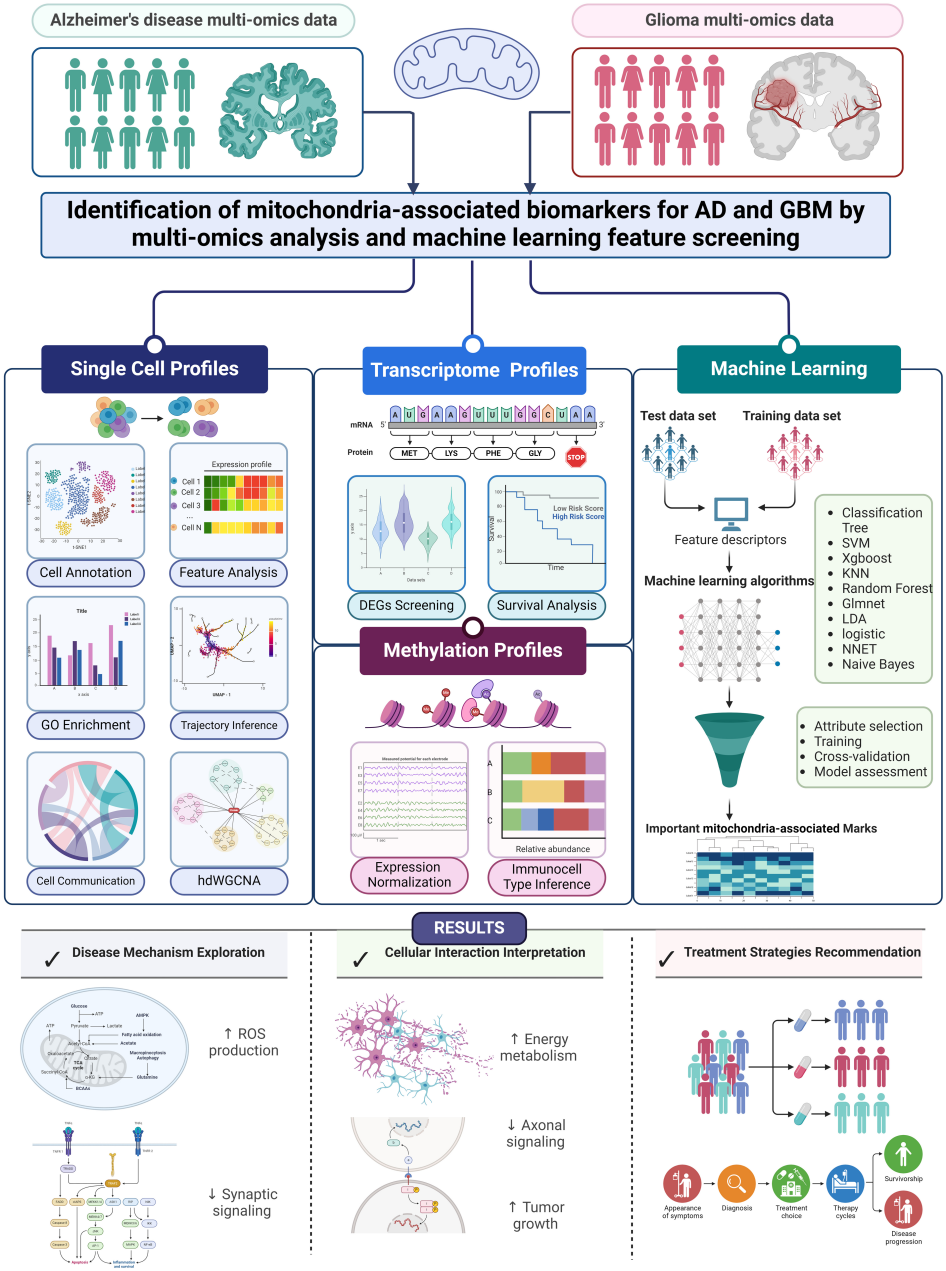
Workflow of unveiling mitochondrial mechanisms in AD and GBM through single-cell transcriptomics and machine learning analysis. Our research aims to bridge gaps in our understanding of Alzheimer’s Disease (AD) and Glioblastoma Multiforme (GBM) by employing single-cell RNA sequencing (scRNA-seq) alongside machine learning techniques. Our approach focuses on identifying biomarkers that exhibit varying expression levels across different types of cells in these conditions. We aimed to find mitochondrial biomarkers for both diseases using 10 machine learning methods, adding to scientific knowledge. Our study used multi-omics data, including RNA sequencing from brain samples of people with AD (*n* = 48) and from GBM patients (*n* = 16). We also included DNA methylation and transcriptome data from large studies. Data access was provided by four major studies: ADNI, ROSMAP, TCGA, and CGGA. We also used data from the GEO database. Informed consent was obtained from all participants, ensuring ethical standards and privacy protection. We performed a step-by-step bioinformatics process that included data normalization, correcting for batch effects, and analyzing gene expression differences. We also used a machine learning system with 10 different algorithms to identify key biomarkers. These algorithms helped us find important biomarkers related to mitochondria. This comprehensive analysis will elucidate the expression patterns and functions of these genes across different cell types. The integration of machine learning with genomic data provides a robust approach for exploring the complex interplay between mitochondrial function and cell identity. This dual-pronged strategy aims to uncover novel biomarkers and regulatory mechanisms, potentially obscured by traditional bulk tissue analysis methods. Our research will identify specific mitochondrial patterns associated with various cell types, providing a nuanced perspective on AD and GBM. This could open up new possibilities for targeted treatments.

In summary, this study addresses a significant gap in our understanding of AD and GBM by focusing on the mitochondrial mechanisms central to both diseases. Despite extensive research, there remains an incomplete understanding of the cellular and molecular underpinnings of these conditions, particularly regarding mitochondrial function. Our approach integrates scRNA-seq data with machine learning algorithms to identify cell-specific mitochondrial markers across distinct cellular subpopulations. Notably, our findings have highlighted the potential involvement of genes linked to mitochondrial epistasis and localization, which may play pivotal roles in the pathogenesis of AD and GBM. By advancing our understanding of mitochondrial contributions to these diseases, we hope this work will guide the development of more targeted and effective therapies for AD and GBM patients. A detailed description of our methodology and the analysis process is presented in Figure 1.

## 2 Methods

### 2.1 Study design

In our research, the objective was to delve into a primary exploration of the possible connections between AD and GBM through a multi-omics lens, with a specific emphasis on mechanisms related to mitochondria. It was our intention to detect potential mitochondrial biomarkers for both conditions by employing a set of 10 machine learning techniques, with the aspiration to augment the current repository of scientific knowledge. The multi-omics data framework for our study encompassed Single-nucleus RNA sequencing (snRNA-seq) profiles from the prefrontal cortex of individuals exhibiting high and low AD pathology (*n* = 48), along with Single-cell RNA sequencing (scRNA-seq) from GBM patients and control samples (*n* = 16). This was complemented by DNA methylation data and comprehensive transcriptome information (ROSMAP: *n* = 740, ADNI: *n* = 1,706, TCGA: *n* = 159, CGGA: *n* = 151). The characteristics of the cohorts for ADNI, ROSMAP, TCGA and CGGA transcriptome and methylation data are listed in Supplementary Table S1. Access to this data was facilitated through the contributions of four esteemed cohort studies: the Alzheimer’s Disease Neuroimaging Initiative (ADNI) (Petersen et al., 2010), the Religious Orders Study and Memory and Aging Project (ROSMAP) (Bennett et al., 2018), which primarily include participants with sporadic late-onset Alzheimer’s Disease (AD); The Cancer Genome Atlas (TCGA),^1^ and the Chinese Glioma Genome Atlas (CGGA)^2^ (Zhao et al., 2021), both of which focus on providing a comprehensive genomic characterization of cancers, emphasizing sporadic cases to capture broader population trends. Additionally, we incorporated datasets from the Gene Expression Omnibus (GEO) series GSE138794 (Wang et al., 2019) and GSE162631 (Xie et al., 2021). For all participants included from the aforementioned studies, informed consents were secured, with unwavering commitment to ethical conduct and the protection of privacy and consent integrity. To bolster the thoroughness of our analytical approach, we engineered a sequential bioinformatics pipeline. This pipeline was designed to encompass processes such as data normalization, correction for batch effects, cellular deconvolution, and the analysis of differential expression. Further, we engaged an integrative machine learning framework, comprising 10 distinct algorithms: Classification Tree, Support Vector Machine (SVM), Xgboost, K-Nearest Neighbors (KNN), Random Forest, Glmnet, Linear Discriminant Analysis (LDA), logistic regression, Neural Networks (NNET), and Naive Bayes. This diverse set of algorithms was utilized to pinpoint significant biomarkers associated with mitochondrial functions.

### 2.2 snRNA-seq/scRNA-seq sample collection

For AD samples, post-mortem brain specimens were secured from 48 subjects who participated in the Religious Order Study (ROS) or the Rush Memory and Aging Project (MAP), renowned for their extensive research on aging and dementia. Data aggregation in ROSMAP encompassed clinical records, meticulous post-mortem evaluations, and intricate molecular analysis of the brain tissues. Among these participants, a subset of 24 exhibited high *β*-amyloid levels alongside AD pathological indicators, while the remaining 24 demonstrated negligible or no β-amyloid presence or related pathological traits. The prefrontal cortex, specifically Brodmann area 10, which is known for its role in cognitive functions often affected by AD, was examined for each subject. Immunohistochemistry confirmed the presence of β-amyloid, and microscopic examinations indicated that the integrity of nuclei in AD-affected samples was comparable to those without pathology. This research resulted in an extensive collection of 80,660 snRNA-seq profiles.

Regarding the GSE138794 context, surgical glioma tissues and peripheral blood were collected from patients who underwent operations at UCSF, with proper Institutional Review Board approval and informed consent. Tissue processing for single-cell RNA sequencing (scRNA-seq) followed a standardized protocol. Fresh tissue was subjected to mechanical and enzymatic dissociation using papain in a DNase I-containing solution, followed by filtration and resuspension in PBS. For frozen tissue, nuclei extraction followed the “Frakenstein” protocol as shared by the 10x Genomics community.^3^ Single-cell capture and complementary DNA (cDNA) synthesis were performed using the Fluidigm C1 system and the SMARTer Ultra Low RNA Kit. This was followed by cDNA quantification, dilution, and library preparation using the Nextera XT DNA Library Prep Kit. Agencourt AMPure XP beads were used for purification and size selection of the libraries. The 10X Genomics platform processed live cells and nuclei from both fresh and frozen tissues on the Chromium Single Cell Capture Chip, strictly adhering to the manufacturer’s guidelines for cell capture, reverse transcription, lysis, and library preparation. Sequencing was carried out on the Illumina NovaSeq platform using a 100 base pair paired-end protocol, yielding crucial data for analyzing the tumor microenvironment at the single-cell level. The dataset includes four IDH-wildtype (IDH-WT) GBMs and four IDH-mutant gliomas, providing a balanced representation of these two molecular subtypes. This composition is crucial for studying the molecular heterogeneity between IDH-WT and IDH-mutant gliomas, given their distinct biological behaviors and clinical outcomes.

For the GSE162631 dataset, surgical specimens were collected from four patients diagnosed with GBM with the aim of isolating endothelial cells (ECs) for single-cell RNA sequencing (scRNA-seq). From each patient, two tissue samples were processed: one extracted from the tumor epicenter and another from the peritumoral region. Tissue dissociation was initiated within 2 h of resection at the research facility to ensure cell viability and integrity. Single-cell suspensions were prepared following mechanical dissociation of the tissues, and specific kits from Miltenyi Biotec were utilized for the processing of tumor and peritumoral tissues. Cellular debris and red blood cells were meticulously removed according to the established protocol. Endothelial cells were then enriched through CD31+ selection using Dynabeads, a magnetic bead-based technology. The samples were subsequently resuspended in Dulbecco’s Phosphate-Buffered Saline (DPBS) with Bovine Serum Albumin (BSA) to facilitate cell counting and viability assessment. The optimal volume for scRNA-seq was selected based on the established protocol to ensure high-quality sequencing results. This dataset consists exclusively of IDH-WT samples, providing insights into the aggressive nature of this subtype, which is associated with a poorer prognosis and limited therapeutic options.

### 2.3 snRNA-seq/scRNA-seq data processing

snRNA-seq/scRNA-seq libraries were crafted employing Chromium Single Cell Reagent Kit (10X Genomics). Subsequently, these libraries were consolidated and subjected to sequencing on the NovaSeq 6,000 platform by Illumina, guided by the NovaSeq Control Software (v1.6.0). The unprocessed sequence data underwent refinement through the Cell Ranger software (v.7.1.0) which enabled the alignment of reads to the human reference genome GRCh38. Consequently, for each specimen, a gene count matrix was formulated. The raw count data of ROSMAP snRNA-seq datasets and scRNA-seq datasets (GSE162631 and GSE138794) were analyzed utilizing the “Seurat” R package (v4.3.0.1). The filtration process was initially conducted to remove cells of poor quality, defined by a total RNA feature count below 200 or mitochondrial RNA surpassing 5%. Following the normalization of cellular gene expression, the SCTransform integration workflow was applied to integrate data from the various datasets. A total of 3,000 anchors for integration were identified, representing close neighbors within the individual datasets. Data scaling was performed to neutralize the effects of the cell cycle and to adjust for the overall counts recorded per cell and the mitochondrial gene expression ratio per cell. Principal Component Analysis (PCA) was then implemented to reduce the data dimensions, yielding 30 principal components. This was followed by the application of t-distributed stochastic neighbor embedding (tSNE) and uniform manifold approximation and projection (UMAP) algorithms for further dimensionality reduction across the initial 15 dimensions. The “doubletFinder” R package (McGinnis et al., 2019) was employed to sift out any doublets from the dataset. The application of the FindNeighbors and FindClusters functions, set at a resolution of 0.5, resulted in the identification of 8 and 12 distinct cell clusters for AD and GBM, respectively. The Wilcoxon rank-sum test was subsequently utilized to pinpoint genes with significant differential expression within each cluster, using the “FindAllMarkers” function with a minimum percentage cutoff of 0.25 and a logarithmic fold change threshold set at 0.25. Finally, the process of cluster consolidation and further exploration of cellular trajectories was facilitated by the “SCP” R package. This package was instrumental in the analysis of differentially expressed genes, cell type annotation, and the elucidation of cellular developmental paths, providing a comprehensive view of the cellular dynamics within the context of AD and GBM.

### 2.4 Cell–cell communication analysis

In multicellular organisms, effective communication between cells is essential for their proper functioning, enabling the sharing of information through the release of signaling molecules or via direct cell-to-cell contact. “CellChat” is a specialized bioinformatics tool designed to infer and quantitatively analyze networks of intercellular communication from scRNA-seq data (Jin et al., 2021). It incorporates comprehensive ligand-receptor interaction databases and is accessible online at http://www.cellchat.org/. This tool facilitates the identification and quantification of interactions based on differential expression levels of ligands and receptors within cell groups, with statistical significance defined by a *p*-value threshold of less than 0.05. The netVisual_diffInteraction function within CellChat was utilized to visually represent variations in the strength of intercellular communication, highlighting the differences between distinct cell populations. To elucidate the complex coordination among multiple cell populations and the signaling pathways that drive intercellular communication, non-negative matrix factorization (NMF) is implemented through the identifyCommunicationPatterns function, which aids in deducing the underlying communication patterns. Subsequently, CellChat enables the extraction of key signaling inputs and outputs among various cell clusters, which can be visually depicted using scatter plots for a clear representation of intercellular signaling dynamics. By measuring the Euclidean distance between pairs of shared signaling pathways on a two-dimensional manifold scatter plot, the rankSimilarity function was applied to pinpoint pathways that exhibit the most significant alterations, particularly in the context of AD and GBM samples. Finally, CellChat allows for the comparison of communication probabilities between ligand-receptor pairs that are regulated by specific cell populations and directed towards others. This comparison is achieved by adjusting the compare parameter within the netVisual_bubble function, providing a nuanced view of cell population-specific regulatory effects within the intercellular communication network. This approach offers valuable insights into the intricate mechanisms of cell–cell interactions in the context of scRNA-seq data analysis.

### 2.5 Integrative machine learning model construction and feature importance selection

The “Boruta” R package is an advanced ensemble method for feature selection, designed to identify the most influential predictors in complex datasets. By employing a *p*-value threshold of 0.01, we enforced a rigorous criterion for variable retention, ensuring that only those features with a statistically significant association to the outcome variable are considered. The algorithm operate by comparing the importance of each feature against a set of randomly generated “shadow” features. This comparison is grounded in the mean decrease in accuracy, a measure that reflects the predictive relevance of each feature. Through iterative evaluation, Boruta distinguishes between truly important features and those that exhibit only spurious correlations. Upon completion of the Boruta process, a subset of “Confirmed” variables was selected—those that have demonstrated a substantial impact on model accuracy and have surpassed the predefined significance threshold. These variables form the basis for subsequent machine learning modeling, streamlining the dataset to its most informative elements. To account for potential confounding factors, such as age, gender, and IDH mutation status, we incorporated these variables as covariates in our machine learning models. This adjustment ensures that the predictive power of the identified gene signatures is evaluated independently of these clinical variables.

For models construction, we adopted a meticulous analytical approach to construct predictive models and identify key biomarkers using the “mlr3” R package.^4^ This package is part of the “mlr3verse,” a comprehensive ecosystem for machine learning in R, known for its flexibility and extensive range of algorithms. Our methodology commenced with a rigorous data preprocessing phase, which included data cleaning to handle missing values and outliers, and normalization to ensure that all features were on a comparable scale. An initial feature selection was conducted based on statistical measures such as variance and correlation, thereby establishing a solid foundation for model training.

The machine learning algorithms were categorized based on their analytical strengths:

- Tree-based models like Classification Tree, Random Forest, and Xgboost, known for their ability to handle non-linear relationships and interactions between features.
- Discriminant and linear models including Linear Discriminant Analysis (LDA) and Glmnet, which are effective for linearly separable data.
- Instance-based learning with K-Nearest Neighbors (KNN), which relies on the similarity of instances for prediction.
- Kernel-based learning via Support Vector Machine (SVM), which uses kernel functions to transform data into a higher-dimensional space.
- Probabilistic models such as Logistic Regression and Naive Bayes, with the latter noted for its simplicity and effectiveness in high-dimensional spaces.
- Neural Networks were also employed for their capacity to capture complex patterns.

Each algorithm underwent hyperparameter optimization using grid search cross-validation, streamlined by the AutoML features of the “mlr3” package, to identify the most effective hyperparameters. Post model training, variable importance scores were extracted and aggregated to pinpoint potential biomarker candidates. To reinforce the stability of feature selection, a process of stability selection was implemented, ensuring the consistency of important features across various data subsets. The predictive performance of the models was then validated using a held-out test set, thereby confirming the generalizability of our models and the relevance of the identified biomarkers. Subsequently, the models were refined using the selected biomarker subset, thereby enhancing both predictive accuracy and model interpretability. Throughout this process, we maintained stringent documentation standards, detailing every step from data preprocessing to model validation. This included hyperparameter settings, feature selection criteria, and variable importance scores.

### 2.6 Transcriptomic data collection and processing

In this research, 694 glioblastoma patients from the TCGA cohort were included. Clinical data were sourced from TCGA’s published works, as detailed previously. Utilizing the Xena Browser by UCSC, TCGA’s gene expression data from AffyU133a arrays and Illumina HiSeq RNA-seq for lower-grade gliomas (LGGs) and glioblastomas (GBMs) were retrieved from https://tcga-data.nci.nih.gov/. Additionally, the CGGA (Chinese Glioma Genome Atlas) dataset, which comprises 692 cases with gene expression profiles for 24,326 genes along with overall survival data, was accessed through the CGGA website at http://www.cgga.org.cn/. This dataset was generated using the Illumina HiSeq 2000 sequencing platform. Ethical approval for this study was granted by the Institutional Review Boards of Beijing Tiantan Hospital, with all participants providing their written consent. The research was conducted in strict compliance with the guidelines and regulations set forth by the Institutional Review Boards. Gene expression values were initially normalized to fragments per kilobase of transcript per million mapped reads (FPKM), considering the gene length. The “limma” R package was employed for data analysis, applying a fold change threshold of greater than 1.5 and an adjusted *p*-value of less than 0.05 to identify differentially expressed genes (DEGs). The “clusterProfiler” R package (Wu et al., 2021) was then used for functional enrichment analysis of the DEGs, categorizing them by Gene Ontology (GO) terms related to biological processes (BP), molecular functions (MF), and cellular components (CC). Additionally, pathway enrichment analysis was conducted with the “clusterProfiler” package, referencing pathways from the Kyoto Encyclopedia of Genes and Genomes (KEGG), with a false discovery rate (FDR) threshold of less than 0.05. For survival analysis, the “survival” and “survminer” R package was applied, offering an extensive toolkit for time-to-event data analysis. The package’s Coxph and Survfit function was utilized to perform Cox proportional hazards regression, a widely recognized approach to explore the correlation between survival outcomes and multiple predictive variables.

### 2.7 Methylation data collection and processing

In our study, we explored the complex patterns of DNA methylation within a cohort comprising 159 glioma tissue samples. These data were meticulously sourced from the TCGA database, facilitated by the “TCGAbiolinks” R package (Colaprico et al., 2016). The methylation status of the samples was assessed using the Illumina Infinium HumanMethylation 450 platform, which quantifies methylation as *β*-values. These values represent the ratio of methylated cytosines to the total cytosine signal. Our investigation centered on methylation events occurring near CpG islands, including their shores and shelves. These regions are of significant interest due to their role in gene regulation. To ensure the accuracy of our analysis, we meticulously curated the data by excluding CpG sites that were in close proximity to known single nucleotide polymorphisms (SNPs), as identified on the Illumina product support website, and those that exhibited cross-reactivity. This exclusion was critical to prevent any misinterpretation of the methylation status. Additionally, we omitted CpG sites located on the Y chromosome, considering their lack of relevance to our all-female patient cohort. These rigorous filters culminated in a refined dataset comprising 180,758 CpG sites, which was then prepared for detailed analysis.

For the samples from the Chinese Glioma Genome Atlas (CGGA), immediate post-surgical snap-freezing in liquid nitrogen was employed to preserve tissue integrity. Hematoxylin and eosin-stained sections were meticulously examined to ensure that only samples with a tumor cell content exceeding 80% were included in the study. Genomic DNA extraction was conducted using the QIAamp DNA Mini Kit, strictly following the manufacturer’s protocol. The purity and concentration of the extracted DNA were determined using a NanoDrop ND-1000 spectrophotometer. The DNA methylation data underwent a series of normalization and processing steps using the ChAMP R package, with its default parameters applied to ensure analytical consistency. Subsequently, the “EpiDISH” R package (Teschendorff et al., 2017) was utilized to deconvolute the methylation profiles. This approach provided an estimation of the relative proportions of various immune cell types within the GBM samples. Our analysis not only deepened the understanding of the immune microenvironment in GBM but also provided valuable insights that could potentially inform the development of targeted immunotherapeutic strategies.

### 2.8 High-dimensional weighted gene co-expression network analysis

In our study, we employed high-dimensional weighted gene co-expression network analysis (hdWGCNA) to delve into the complex relationships within single-cell RNA sequencing (scRNA-seq) data. This approach was facilitated by the “hdWGCNA” R package (Morabito et al., 2023), which is specifically designed to create gene correlation networks suited for scRNA-seq data. We initiated our analysis by creating a Seurat object employing the SetupForWGCNA function. To counteract the sparsity characteristic of scRNA-seq data, we constructed metacells, which are clusters of transcriptionally similar cells, using the k-Nearest Neighbors (KNN) algorithm with default parameters: *k* = 25 for the number of neighbors and max_shared = 10 to control the sharing of cells between metacells. The determination of the optimal soft power threshold for gene–gene correlation scaling was performed using the TestSoftPowers function, which was set to explore a range of soft power values from 1 to 30 to achieve the best fit to the scale-free topology model. The ConstructNetwork function was then utilized to create a topological overlap matrix (TOM) for module detection, employing the scaled gene–gene correlations. Module dendrograms were visualized using the PlotDendrogram function to provide a clear hierarchical representation of gene clusters. Module eigengenes (ME), calculated with the ModuleEigengenes function using default settings, served as the first principal components of each module’s gene subset. Intra-modular connectivity was quantified using the SignedKME algorithm, which determined the kME metric for each gene in relation to its module’s eigengene. This step was crucial for identifying the most interconnected genes within each module. For dimensionality reduction and visualization, the RunModuleUMAP function was applied to the TOM, focusing on the top five hub genes per module as ranked by their kME values. This resulted in a UMAP visualization that was predominantly influenced by these hub genes, with annotations for the top five hub genes in each module to reflect the module’s key features.

### 2.9 Statistical analyses

All statistical analyses and data visualizations were performed using R software (version 4.3.3). Differences in survival between the two groups were assessed using Kaplan–Meier curves and the log-rank test. Univariate and multivariate Cox regression analyses were used to determine prognostic factors. For correlation analysis, correlation coefficients were calculated using Pearson for normally distributed data and Spearman for non-normally distributed data. For analysis of differences between two groups of data, unpaired Student’s t-test and Mann–Whitney U-test were used for normally and non-normally distributed variables, respectively. To compare more than two groups, one-way analysis of variance (ANOVA) and Kruskal–Wallis’s tests were used as parametric and nonparametric methods, respectively. The adjusted *p*-value (FDR) was calculated by the Benjamini–Hochberg correction method. *p* < 0.05 was considered statistically significant unless mentioned otherwise.

## 3 Results

### 3.1 Single-cell transcriptomics unveils distinct mitochondrial signatures and cellular heterogeneity in AD and GBM

**Figure 2:**
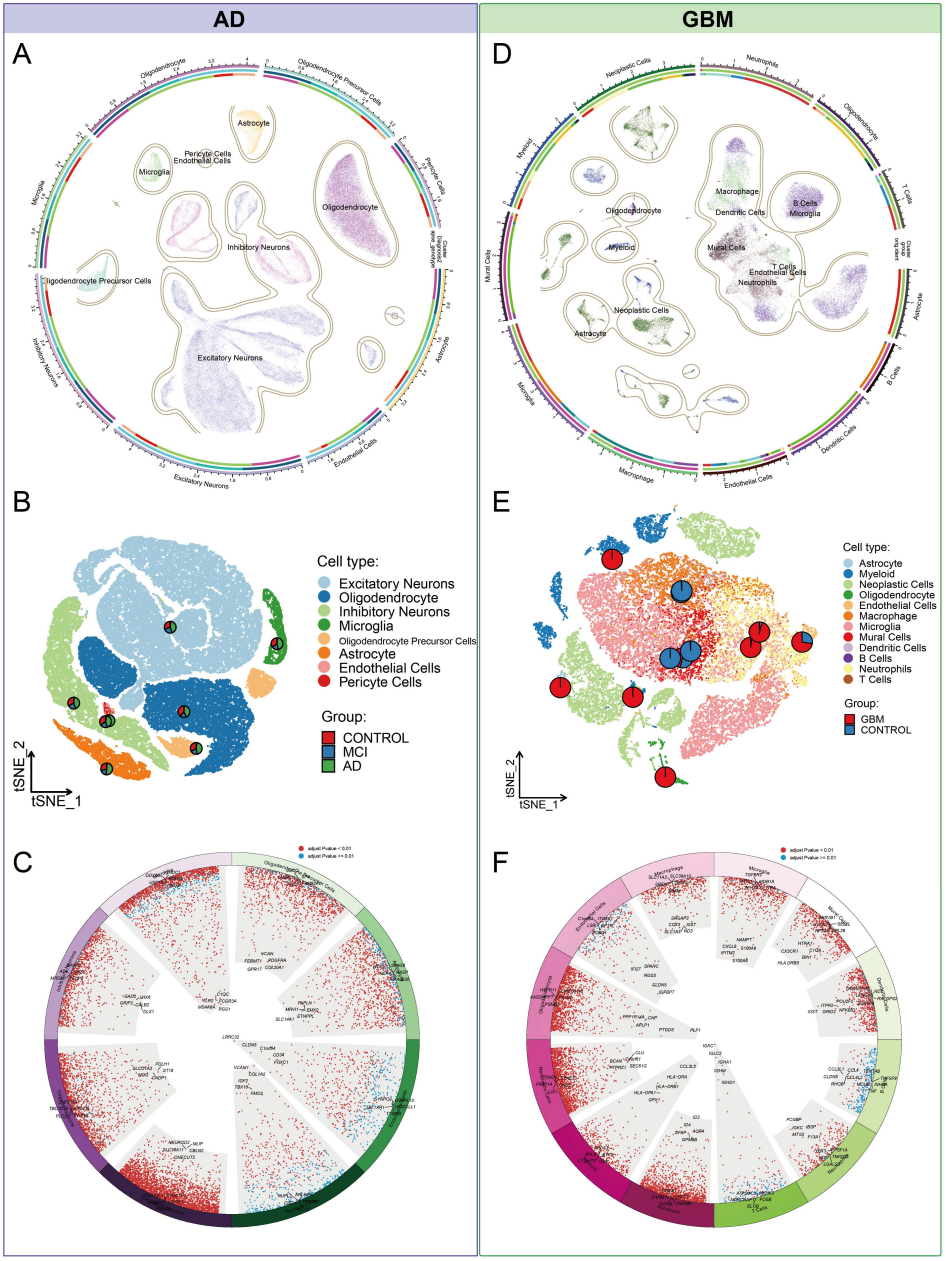
Cellular subpopulation landscape in AD and GBM. **(A–C)** Our investigation of AD utilized single-nucleus RNA sequencing (snRNA-Seq) to meticulously annotate and delineate cellular subpopulations in relation to established biological processes. Post stringent quality control measures, a dataset of 70,634 cells was subjected to dimensionality reduction techniques, such as Uniform Manifold Approximation and Projection (UMAP) and t-Distributed Stochastic Neighbor Embedding (tSNE), coupled with cluster analysis. This approach identified eight distinct cellular subpopulations, including Oligodendrocytes (25.8%), Inhibitory Neurons (13.0%), Excitatory Neurons (4.9%), Astrocytes (4.8%), Oligodendrocyte Precursor Cells (OPCs, 3.7%), Microglia (2.7%), Pericytes (0.2%), and Endothelial Cells (0.2%). **(D–F)** To explore the heterogeneity of GBM, we integrated single-cell RNA sequencing (scRNA-seq) data from two distinct datasets (GSE138794 and GSE162631). The SCTransform algorithm was applied to mitigate batch effects, resulting in an integrated dataset comprising 35,674 cells across 16 samples. Unsupervised clustering analysis identified 38 distinct clusters, which were categorized into 12 cell types: Microglia (35.6%), Neoplastic Cells (22.3%), Neutrophils (11.9%), Macrophages (10.2%), Myeloid Cells (8.1%), Mural Cells (6.5%), Dendritic Cells (1.8%), Oligodendrocytes (1.7%), Endothelial Cells (1.1%), Astrocytes (0.4%), T Cells (0.2%), and B Cells (0.1%). This comprehensive categorization facilitates a deeper understanding of the cellular diversity within AD and GBM and paves the way for targeted therapeutic strategies.

**Figure 3:**
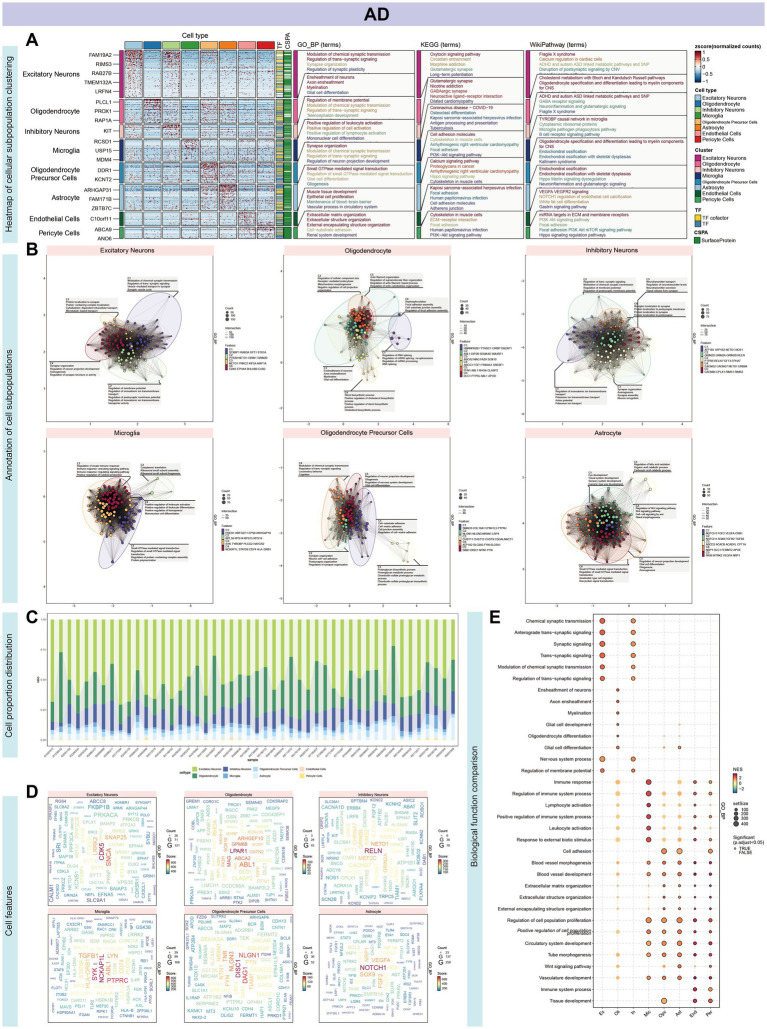
Characterization and functional annotation of AD single-cell subpopulations. **(A)** Hierarchical clustering, utilizing the Wilcoxon rank sum test, was employed to identify differentially expressed genes (DEGs) within each cluster, revealing a substantial representation of nerve cells. **(B)** Functional enrichment analysis revealed unique gene sets for each cell subset. Excitatory Neurons predominantly expressed genes linked to chemical synaptic transmission modulation and trans-synaptic signaling regulation. DEGs from cellular subpopulations, particularly the C2 cluster marked by *ABL1, DIP2B, SEMA4D*, and *SMURF1* in Oligodendrocytes, showed significant enrichment in the mitochondrial morphogenetic pathway. **(C)** The histogram illustrates the proportional representation of cell types within each sample. **(D)** Cell subpopulations were characterized by defining genes: Excitatory Neurons (*CDK5*), Oligodendrocytes (*LPAR1*), Inhibitory Neurons (*RELN*), Microglia (*NCKAP1L*), OPCs (*DISC1*), and Astrocytes (*NOTCH1*). Notably, *LPAR1, RELN*, and *NCKAP1L*, implicated in mitochondrial epistasis, and *DISC1*, a gene associated with mitochondrial localization, were identified. This underscores the complex interplay between cellular identity and mitochondrial function in neurological diseases. **(E)** GSVA enrichment analysis across cell types revealed heightened involvement in signal transduction within the nervous system, including synaptic transmission, as well as processes related to cell growth and differentiation.

Our investigation began with single-nucleus RNA sequencing (snRNA-Seq) of AD, followed by comprehensive data annotation to delineate cellular subpopulations and their associated biological processes. After stringent quality control, we obtained a dataset comprising 70,634 cells for analysis. Using dimensionality reduction techniques, including UMAP and tSNE, alongside clustering analyses, we identified eight distinct cellular subpopulations: Oligodendrocytes (25.8%), Inhibitory Neurons (13.0%), Excitatory Neurons (4.9%), Astrocytes (4.8%), Oligodendrocyte Precursor Cells (OPCs, 3.7%), Microglia (2.7%), Pericytes (0.2%), and Endothelial Cells (0.2%) (Figures 2A–C). Hierarchical clustering and the Wilcoxon rank sum test were utilized to detect DEGs within each cellular cluster, revealing a significant representation of nerve cells. Each cell type exhibited a unique set of functionally distinct genes. For instance, Excitatory Neurons predominantly expressed genes associated with the modulation of chemical synaptic transmission and the regulation of trans-synaptic signaling processes. Subsequent clustering and enrichment analysis of DEGs from these cellular subpopulations uncovered that the C2 cluster, characterized by genes *ABL1, DIP2B, SEMA4D,* and *SMURF1* in Oligodendrocytes, was significantly enriched for the mitochondrial morphogenetic pathway (Figures 3A,B). The defining genes of each cell subpopulation, as determined through cellular profiling, were as follows: Excitatory Neurons (*CDK5*), Oligodendrocytes (*LPAR1*), Inhibitory Neurons (*RELN*), Microglia (*NCKAP1L*), OPCs (*DISC1*), and Astrocytes (*NOTCH1*) (Supplementary Table S2). Notably, *LPAR1, RELN*, and *NCKAP1L* are associated with mitochondrial epistasis, genes previously identified in our study, while *DISC1* is recognized as a gene related to mitochondrial localization (Figures 3C,D).

**Figure 4:**
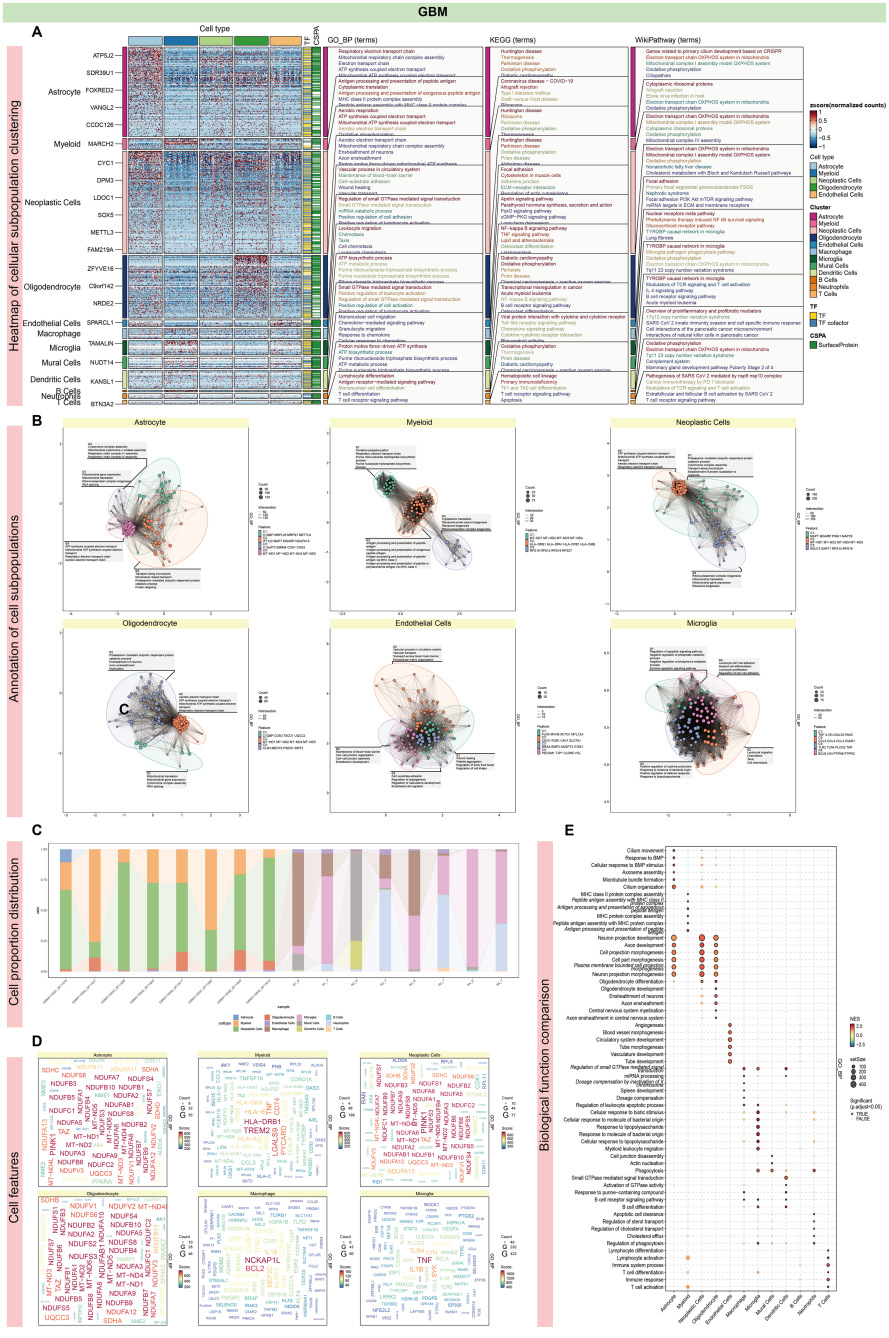
Characterization and functional annotation of GBM single-cell subpopulations. **(A)** Differential gene expression analysis identified Neoplastic Cells exhibited the highest frequency of DEGs, followed by Astrocytes and Oligodendrocytes. **(B)** Functional enrichment analysis of different cell subsets. All three cell types showed significant enrichment in mitochondria-related processes, including mitochondrial gene expression and mitochondrial ATP synthesis coupled with electron transport. They commonly had enrichment for mitochondrial gene clusters including *MT-ND1, MT-ND2, MT-ND4,* and MT-ND5. The differentially expressed genes in these cells included numerous genes coupled with the NADH dehydrogenase (ubiquinone) 1 beta subcomplex, a key component of the mitochondrial respiratory chain complex I. **(C)** The histogram illustrates the proportional representation of cell types within each sample. **(D)** Mitochondrial genes in these cell types were significantly enriched for processes related to mitochondrial function, including gene expression and electron transport-coupled ATP synthesis, underscoring their role in energy metabolism. **(E)** This enrichment highlights the intimate connection of these cell types with mitochondrial function and energy metabolism. Additionally, enrichment for processes involved in the development of neuronal and cellular projections was observed, indicating a role in cellular and neural development. Our single-cell analysis outcomes from AD and GBM highlight shared involvement in key biological processes. Both conditions show significant enrichment in mitochondria-related processes, particularly oxidative phosphorylation, and pathways influenced by small GTPases. Immune-related processes are also commonly enriched, with a notable role for microglia. Oligodendrocyte function is conserved in both diseases, closely associated with mitochondrial processes such as morphogenesis and energy metabolism, as well as axon ensheathment and neuronal morphogenesis. KEGG disease enrichment analysis of GBM’s oligodendrocyte signature genes suggests correlations with other neurodegenerative conditions, including Huntington’s disease, Parkinson’s disease, and AD. AD exhibits heightened enrichment in nervous system signaling, including synaptic transmission, and processes related to cell growth and differentiation. In contrast, GBM is characterized by a greater emphasis on mitochondrial energy metabolism, evident in the coupling of ATP synthesis with electron transport and the ATP biosynthetic process. Furthermore, GBM shows significant enrichment in immune response processes, particularly lymphocyte and T cell differentiation.

To investigate GBM heterogeneity, we conducted an integrated analysis of scRNA-seq data from two distinct datasets (GSE138794 and GSE162631). The SCTransform algorithm was utilized to mitigate batch effects, resulting in an integrated dataset comprising 35,674 cells across 16 samples. UMAP analysis identified 38 clusters, categorized into 12 cell types according to existing annotations: Microglia (35.6%), Neoplastic Cells (22.3%), Neutrophils (11.9%), Macrophages (10.2%), Myeloid Cells (8.1%), Mural Cells (6.5%), Dendritic Cells (1.8%), Oligodendrocytes (1.7%), Endothelial Cells (1.1%), Astrocytes (0.4%), T Cells (0.2%), and B Cells (0.1%) (Figures 2D–F). Differential gene expression analysis indicated that neoplastic cells exhibited the highest frequency of DEGs, followed by astrocytes and oligodendrocytes (Figure 4A; Supplementary Table S3). These cell types were significantly enriched for mitochondrial processes, including mitochondrial gene expression, electron transport chain activity, and ATP synthesis, with key genes such as *MT-ND1*, *MT-ND2*, *MT-ND4*, and *MT-ND5* prominently expressed (Figures 4B–D). These genes were also linked to the NADH dehydrogenase (ubiquinone) 1 beta subcomplex, a crucial component of mitochondrial complex I, indicating a pivotal role in mitochondrial energy metabolism. Additionally, enrichment analysis showed these cells were involved in neuronal and cellular projections, highlighting their roles in neural development (Figure 4E).

Comparative analyses of single-cell transcriptomics in AD and GBM revealed shared mitochondrial involvement in oxidative phosphorylation, immune response, and small GTPase-related signaling pathways. Both AD and GBM showed significant immune-related enrichments, particularly involving microglia. Notably, oligodendrocytes in both conditions displayed enrichment in mitochondrial processes such as morphogenesis, energy metabolism, axon ensheathment, and neuronal morphogenesis. KEGG pathway enrichment of oligodendrocyte-specific genes in GBM indicated associations with Huntington’s, Parkinson’s, and Alzheimer’s disease. Additionally, AD showed higher enrichment in synaptic transmission and neuronal signaling, whereas GBM emphasized mitochondrial energy metabolism, ATP synthesis, and immune response processes, including T cell differentiation (Figures 3E, 4E).

### 3.2 Decoding complex intercellular networks: cell–cell communication analysis reveals critical signaling pathways in AD and GBM

**Figure 5:**
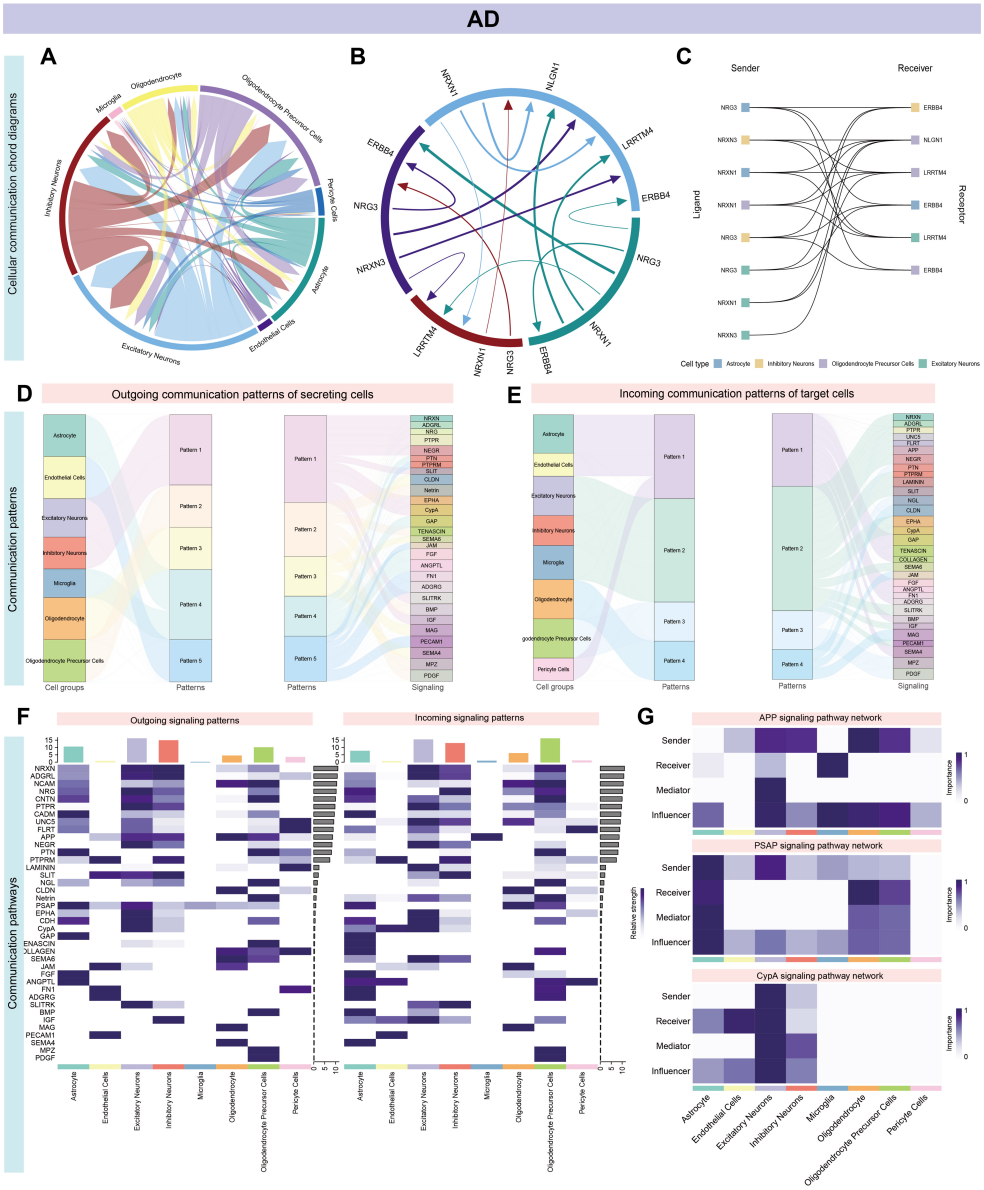
Systems analysis of cell–cell communication network in AD single-cell data. Panel **(A–G)** and Supplementary Figure S1 illustrate the intricate signaling networks among various cell types in the brains of patients with AD, as revealed by cell communication analysis. **(A,B)** The aggregated cell–cell communication network highlights prominent receptor-ligand interactions, especially among neuronal cells such as OPCs, Astrocytes, Excitatory Neurons, Inhibitory Neurons, and Oligodendrocytes. Significant interactions are observed between OPCs and Astrocytes, as well as between Excitatory and Inhibitory Neurons. **(C)** The most impactful pathways are ranked by the number of interactions, with ADGRL (27), PTPRM (25), UNC5 (23), NRXN and PSAP (each with 23), and FLRT (21) leading the list. Within Astrocytes, Excitatory/Inhibitory Neurons, and OPCs, the ligand-receptor pairs NRG3-ERBB4, NRXN3-LRRTM4, and NRXN1-NLGN1 stand out (Supplementary Figures S1B,C). **(D,E)** Signal classification into outgoing and incoming facilitates pattern recognition and cluster analysis, identifying five distinct efferent and four afferent signaling patterns. **(F)** Excitatory and Inhibitory neurons show similar conduction patterns, with the ADGRL and NCAM pathways being the primary routes for signal secretion. **(G)** Network centrality scores for the APP, PSAP, and CypA signaling pathways are computed and visualized, indicating their central roles in the cellular communication network.

Cell communication analysis revealed intricate signaling networks among various brain cell types in AD and GBM. In AD, distinct receptor-ligand interactions were identified, prominently involving OPCs, astrocytes, excitatory and inhibitory neurons, and oligodendrocytes. The strongest interactions were observed between OPCs and astrocytes, as well as between excitatory and inhibitory neurons (Figures 5A,B; Supplementary Figure S1A; Supplementary Table S4). The five most significant signaling pathways were *ADGRL* (27 interactions), *PTPRM* (25 interactions), *UNC5* (23 interactions), *NRXN* (23 interactions), and *PSAP* (21 interactions) (Figure 5C; Supplementary Figures S1B,C; Supplementary Table S5). Notably, the ligand-receptor pairs *NRG3-ERBB4*, *NRXN3-LRRTM4*, and *NRXN1-NLGN1* exhibited the most substantial interactions across astrocytes, excitatory/inhibitory neurons, and OPCs (Supplementary Figures S1B,C). Outgoing and incoming signal patterns were categorized for cluster analysis, revealing five efferent and four afferent patterns, with *ADGRL* and *NCAM* pathways dominating secreted signals in excitatory and inhibitory neurons (Figures 5D–F). Astrocytes and oligodendrocytes exhibited distinct secretion patterns, enriched for pathways such as *APP* and *PTPR* (Supplementary Figure S1C).

**Figure 6:**
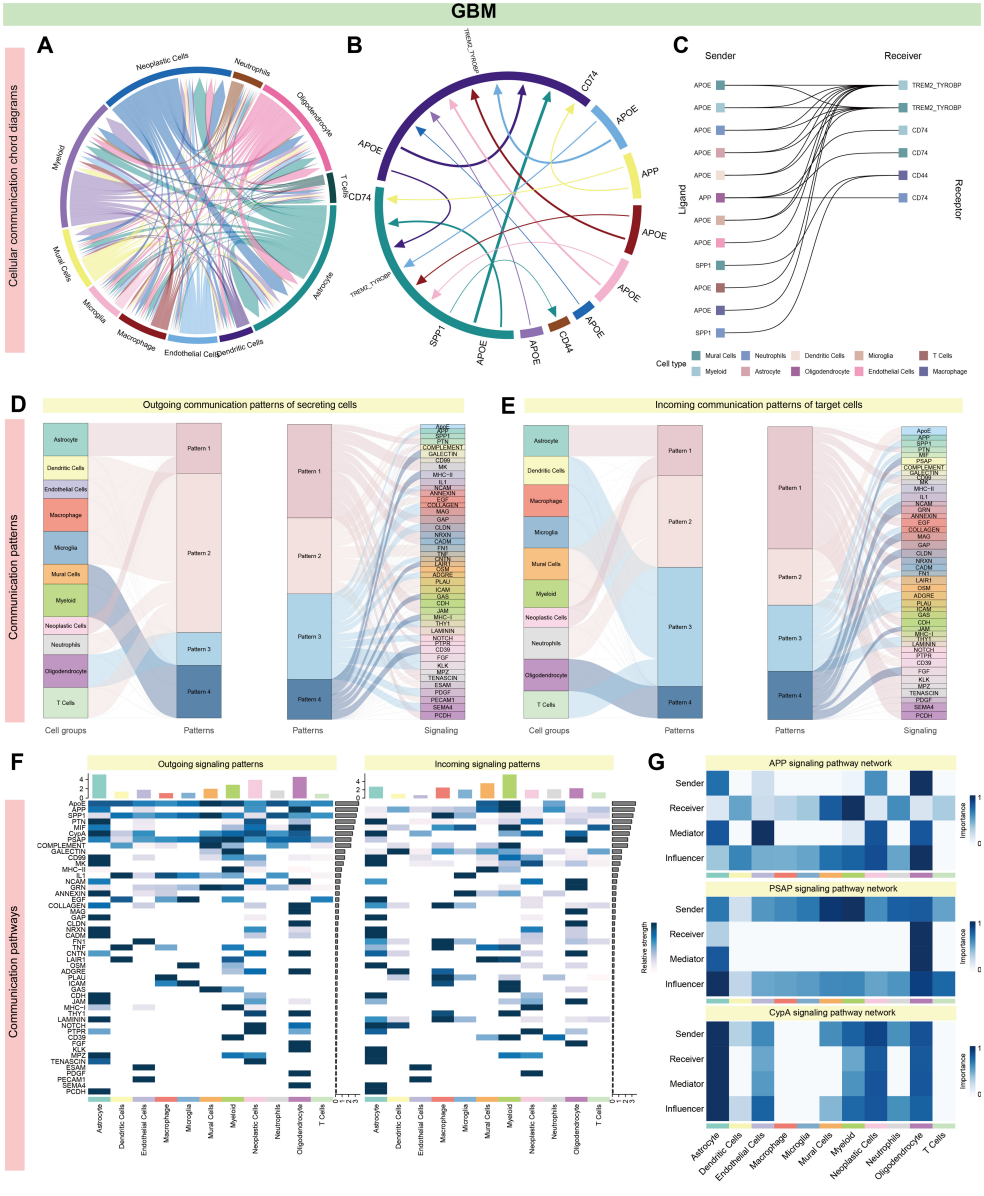
Systems analysis of cell–cell communication network in GBM single-cell data. **(A,B)** In GBM, Astrocytes, Myeloid Cells, Neoplastic Cells, and Oligodendrocytes exhibit particularly prominent intercellular interactions, as evidenced by high interaction counts (Astrocytes: 106 and 88, Myeloid Cells: 75 and 97, Neoplastic Cells: 100 and 78, Oligodendrocytes: 77 and 83). The most substantial interactions occur between Astrocytes and Neoplastic Cells, with Oligodendrocytes also showing significant interactions with both Astrocytes and Neoplastic Cells, each with 14 interactions. This suggests a key role for these cell types in the GBM cellular communication network. **(C)** A significant enrichment of the APOE or APP pathway is noted across various cell types, including Astrocytes, Oligodendrocytes, Microglia, Dendritic Cells, Macrophages, and T cells. Notably, the APP-CD74 and APP-(TREM2 + TYROBP) interactions constitute the majority of these pathway interactions. This prevalence suggests a central role for the APP pathway in the cellular interactions in GBM, potentially influencing disease progression and response to treatment (Supplementary Figures S2B,C). **(D–F)** All cell types display signaling patterns categorized into four distinct outgoing and four incoming patterns. Microglia and certain immune cells, such as Dendritic Cells, Macrophages, and T cells, exhibit similar signaling patterns, suggesting a coordinated immune response. In contrast, Astrocytes and Oligodendrocytes have unique transmission patterns, but both are enriched for the APP pathway, indicating a distinct role for these glial cells in disease pathology, potentially affecting tumor growth and therapy response [Figure 5G and panel **(G)**]. A total of 39 signaling pathways are enriched in AD, while 49 are enriched in GBM, with 23 pathways common to both. The APP pathway is the most significant, accounting for 24.7% of total interactions (705 out of 2,851), followed by the PSAP pathway, which represents 16.2% of interactions (462 out of 2,851). Other notable pathways include CypA, PTN, and NRNX, highlighting the APP pathway’s potential as a central mechanism in disease pathology.

In GBM, intercellular interactions were predominantly centered around astrocytes, myeloid cells, neoplastic cells, and oligodendrocytes. The highest interaction frequency was noted between astrocytes and neoplastic cells (18 interactions), followed by oligodendrocytes and astrocytes (Figures 6A,B; Supplementary Figure S2A; Supplementary Table S5). The *APOE* and *APP* pathways were significantly enriched across multiple cell types, including astrocytes, oligodendrocytes, microglia, dendritic cells, and macrophages. Among these, *APP-CD74* and *APP-(TREM2 + TYROBP)* accounted for the largest proportion of interactions (Figure 6C; Supplementary Figures S2B,C; Supplementary Table S5). Cell communication patterns were categorized into four outgoing and four incoming modes, with immune cells (e.g., microglia, dendritic cells, and macrophages) showing similar signaling profiles. Both astrocytes and oligodendrocytes were uniquely enriched for the *APP* pathway (Figures 6D–F and Supplementary Figures S2B,C).

In summary, 39 signaling pathways were enriched in AD and 49 in GBM, with 23 pathways shared between both conditions. The *APP* pathway emerged as the most significant (705/2851, 24.7%), followed by *PSAP* (462/2851, 16.2%), *CypA* (266/2851, 9.3%), *PTN* (252/2851, 8.8%), and *NRXN* (207/2851, 7.2%). Oligodendrocytes were key contributors to the *APP* signaling pathway (Figures 5G, 6G). Supplementary Figures S1, S2 illustrate specific receptor-ligand pairs across key cell types. The central roles of the *NRXN* and *APP* pathways in AD and GBM were highlighted, reflecting their distinct yet overlapping contributions to neuronal and neoplastic cells, respectively (Supplementary Figures S1D, 2D).

### 3.3 Comparative transcriptomics identifies distinct gene expression profiles and functional pathways in AD and GBM

**Figure 7:**
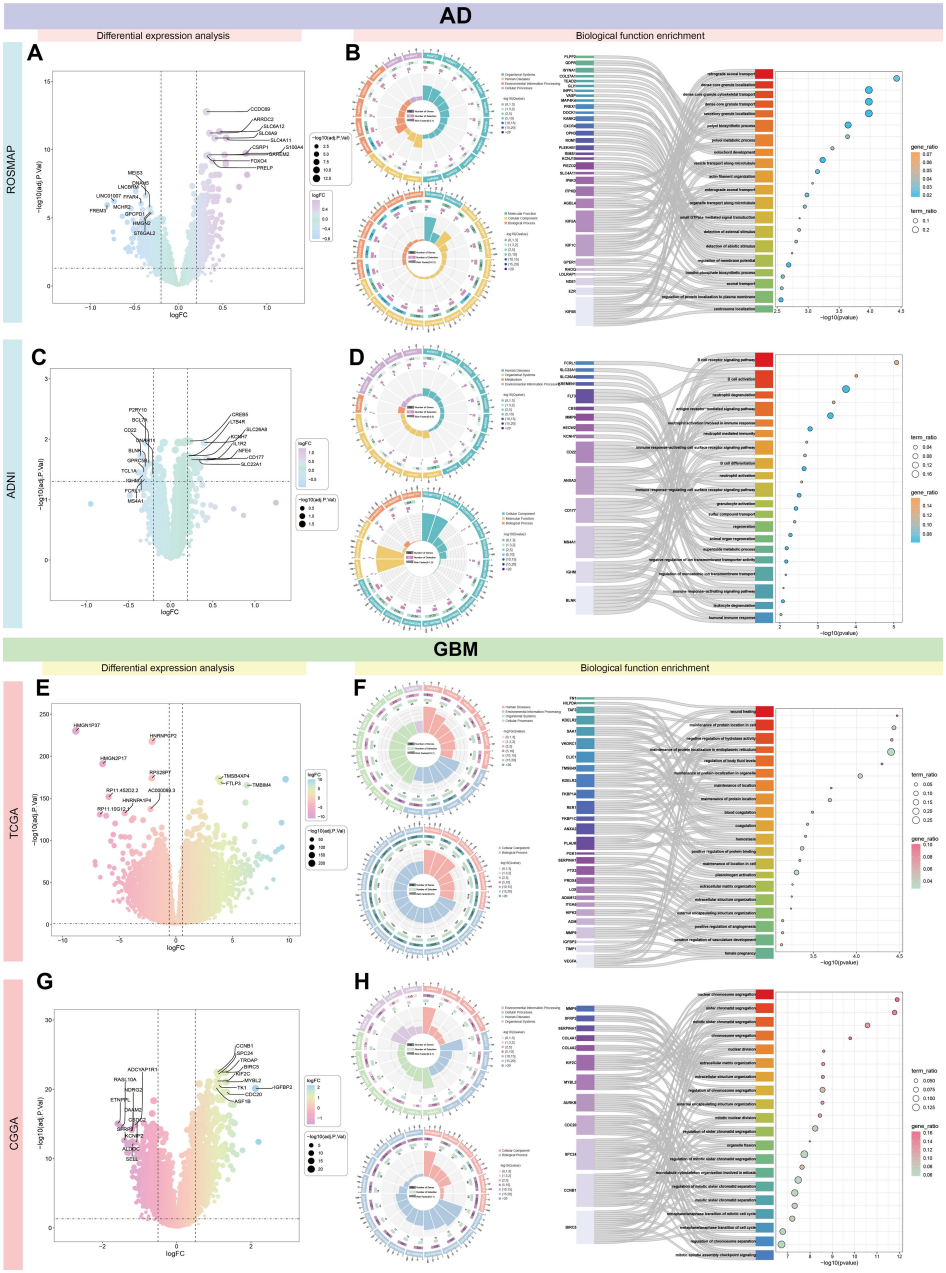
Comprehensive transcriptomic characterization of AD and GBM. This figure presents a comprehensive gene expression analysis across four distinct databases—ROSMAP, ADNI, TCGA, and CGGA—comparing differentially expressed genes in AD and GBM. The analysis highlights a significant variation in the number of up-and down-regulated genes among the datasets: ROSMAP **(A)** displays 436 up-regulated and 1,267 down-regulated genes, ADNI **(C)** shows 25 up-regulated and 11 down-regulated genes, TCGA **(E)** exhibits 5,125 up-regulated and 5,121 down-regulated genes, and CGGA **(G)** shows 84 up-regulated and 33 down-regulated genes. The 10 most significantly up-and down-regulated genes are illustrated for each database, with TCGA demonstrating the most substantial differences in gene expression and ADNI showing the least, suggesting a diverse molecular landscape across patient cohorts. The ROSMAP dataset **(B)** indicates that differential genes are predominantly involved in nuclear chromosome segregation, suggesting roles in cell division and genomic stability. In contrast, the ADNI dataset **(D)** reveals that differential genes are primarily associated with immune-related processes, such as B cell activation and signaling pathways (Supplementary Figures S3A,B,E–F), emphasizing the involvement of immune mechanisms in AD pathogenesis. For GBM, the TCGA **(F)** and CGGA **(H)** datasets show that differential genes are mainly implicated in protein localization and assembly, and chromosome segregation and mitotic processes (Supplementary Figures S3C,D,G–H), indicating a significant impact on tumor growth and progression, which could influence therapeutic strategies. The differential gene expression patterns observed across these databases provide valuable insights into the molecular mechanisms underlying AD and GBM, emphasizing the heterogeneity in disease pathology and the necessity for tailored therapeutic approaches. Understanding these patterns can guide the development of targeted therapies and personalized medicine strategies, ultimately aiming to improve patient outcomes.

Gene expression analysis across four databases identified DEGs with the following up-and down-regulation counts: ROSMAP (up-regulated: 436, down-regulated: 1,267), ADNI (up-regulated: 25, down-regulated: 11), TCGA (up-regulated: 5,125, down-regulated: 5,121), and CGGA (up-regulated: 84, down-regulated: 33). The 10 most significantly up-and down-regulated genes were depicted in Figures 7A,C,E,G. The TCGA dataset demonstrated the most pronounced gene expression differences, whereas the ADNI dataset exhibited the fewest.

Functional analysis of DEGs in the ROSMAP dataset revealed significant enrichment in nuclear chromosome segregation. In contrast, DEGs in the ADNI dataset were primarily associated with immune-related functions, particularly B cell activation and signaling pathways (Figures 7B,D and Supplementary Figures S3A,B,E–H). For GBM, differential gene expression analysis indicated that TCGA genes were enriched in protein localization and assembly, while CGGA genes were largely involved in chromosome segregation and mitotic processes (Figures 7F,H and Supplementary Figures S3C,D,G,H).

When consolidating all differential expression analyses, the most common enrichments were found in cellular processes, binding activities, and cellular anatomical structures. KEGG pathway analysis identified significant shared enrichment across signaling pathways. Genes up-regulated in both AD and GBM were enriched in mitosis, osteoblast morphogenesis, and protein-localized transcriptional maintenance. In contrast, down-regulated genes were most significantly associated with nuclear transcription, synaptic signaling, and calcium ion transport (Supplementary Figure S4).

### 3.4 Integration of machine learning and multi-omics data identifies novel cell-specific mitochondrial markers in AD and GBM

Using differentially expressed genes from each cell type in AD and GBM single-cell datasets, we implemented a screening process utilizing ten distinct machine learning algorithms. To investigate the impact of mitochondria-related genes, we compared mitochondrial epistasis genes with mitochondrial localization genes based on previous studies (Xu et al., 2022, 2024). Our analysis identified 275 cell-specific mitochondrial epistasis genes (MT-Inter) and 94 cell-specific mitochondrial localization genes (MT-Locate) (Supplementary Tables S6, S7). The predictive potential and variable significance of these genes were evaluated using gene expression data from four databases: ROSMAP, ADNI, TCGA, and CGGA. The models were assessed using seven performance metrics: Area Under the Curve (AUC), Confusion Entropy (CE), Accuracy (ACC), Precision, Recall, Sensitivity, and Specificity.

**Figure 8:**
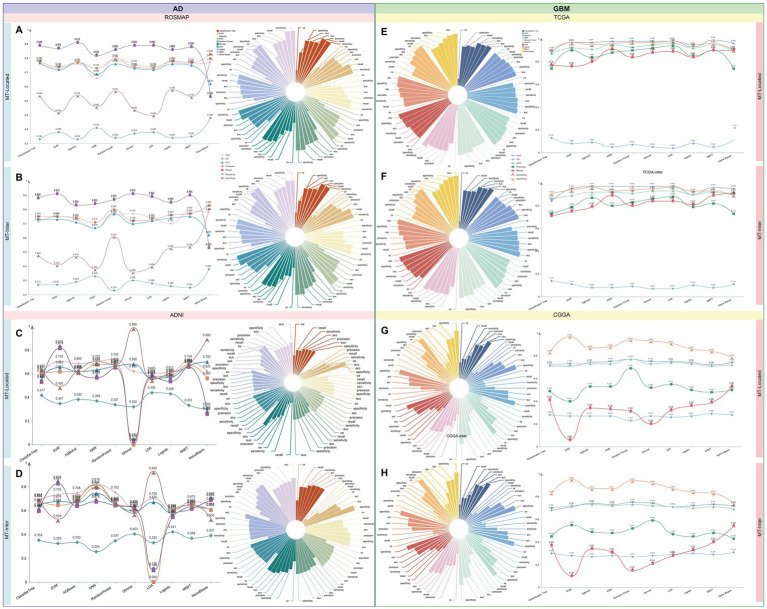
Machine learning-driven identification of mitochondria-associated markers in AD and GBM. We employed a panel of 10 distinct machine learning algorithms to screen differentially expressed genes identified for each cell type. Our analysis contrasted mitochondrial epistasis-related genes and mitochondrial localization genes, building upon our prior research (Xu et al., 2022, 2024). The analysis yielded 275 cell-specific mitochondrial epistasis genes (MT-Inter) and 95 cell-specific mitochondrial localization genes (MT-Locate). The predictive potential and variable significance of these genes were appraised using gene expression data from four databases: ROSMAP, ADNI, TCGA, and CGGA. Model evaluation was conducted through seven metrics, including Area Under the Curve (AUC), Confusion Entropy (CE), Accuracy (ACC), Precision, Recall, Sensitivity, and Specificity. **(A–D)** In AD, the ROSMAP database’s mitochondrial localization genes, as determined by the Random Forest algorithm, showed the highest predictive efficiency (AUC = 0.8). The KNN algorithm, applied to mitochondrial epistasis-related genes in the ADNI database, demonstrated superior predictive performance (AUC = 0.82). **(E–H)** In GBM, the TCGA dataset’s Classification Tree model had an AUC just below 0.9 (TCGA-Locate AUC = 0.88, TCGA-Inter AUC = 0.84), with other models achieving AUCs over 0.95. Notably, the TCGA-Locate model achieved the highest AUC score of 0.99. In the CGGA dataset, the Random Forest-based CGGA-Inter model was the most effective, with an AUC of 0.77. These findings highlight the value of machine learning algorithms in identifying and predicting mitochondria-associated cell-specific markers, offering insights into the molecular foundations of AD and GBM and aiding in the development of targeted therapeutic strategies.

For AD, Random Forest algorithm using mitochondrial localization genes from ROSMAP showed the highest predictive efficiency (AUC = 0.8). In the ADNI database, the KNN algorithm, using mitochondrial epistasis-related genes, achieved the best predictive performance (AUC = 0.82). Focusing on GBM, within the TCGA dataset, the Classification Tree’s AUC fell just below 0.9 (TCGA-Locate AUC = 0.88, TCGA-Inter AUC = 0.84), while all other models showed exceptionally high results, with AUCs surpassing 0.95. Notably, the TCGA-Locate model achieved the highest AUC score of 0.99. In the CGGA dataset, the Random Forest-based CGGA-Inter model emerged as the most effective (AUC = 0.77) (Figure 8 and Supplementary Table S8).

Integrating variable importance from the top three predictive models allowed us to identify key markers within the MT-Inter and MT-Locate categories for each of the four datasets: ADNI (9/5), ROSMAP (25/16), and CGGA (39/49). For the TCGA dataset, the top 50 marker genes were selected. Network analysis revealed significant intersections among gene sets, with the TCGA-Locate and CGGA-Locate models showing the highest number of shared genes (17) (Supplementary Figure S5 and Supplementary Table S9). Notably, *ERBB4* was a significant contributor across RUSH-Locate, CGGA-Locate, TCGA-Locate, and CGGA-Inter models.

By compiling biomarkers of high importance, we curated a list of 24 candidate cell-specific mitochondria-associated markers, each demonstrating significant contributions in at least one dataset for both AD and GBM (Supplementary Table S10). Among them, 16 were classified as mitochondrial epistasis genes, and 13 as mitochondrial localization genes. Five genes, *ERBB4, ABAT, FAM110B, MAPK10*, and *PRKCA*, were classified as both mitochondrial epistasis and mitochondrial localization genes. Their distinct expression patterns across different cell types of AD and GBM were illustrated in Supplementary Figures S6, S7. These candidate marker genes were predominantly expressed in oligodendrocytes and neoplastic cells, with distinct distribution patterns across neural cells in AD and GBM compared to immune cells. Particularly, significant disparities in expression were noted between astrocytes and oligodendrocytes.

### 3.5 Inference of immune cell components based on methylation data

**Figure 9:**
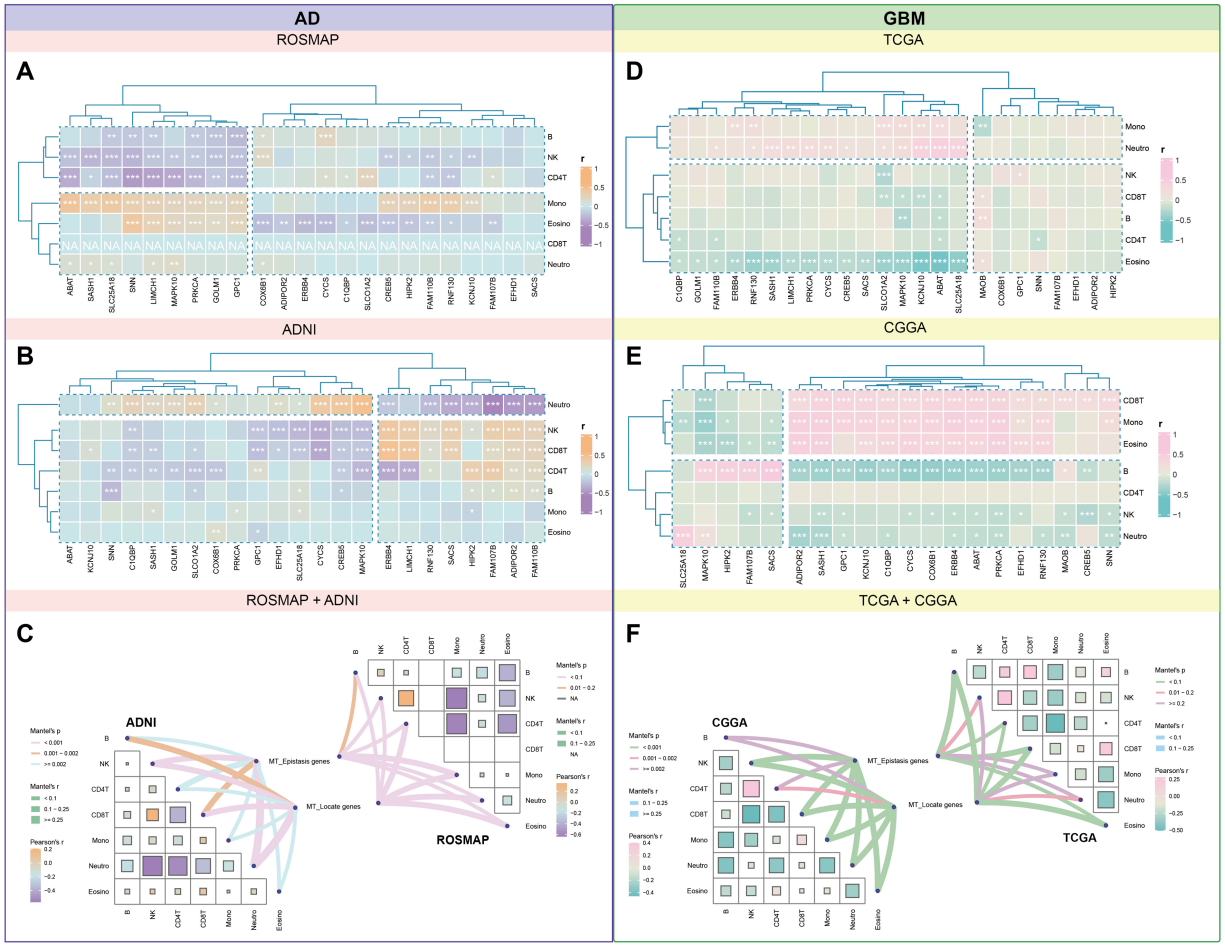
Correlational landscape of immune cell-gene expression in AD and GBM. We conducted an analysis of the intricate interactions between immune cells and the tumor microenvironment (TME) in the context of AD and GBM, as deduced from methylation data. We observed significant correlations between the expression of candidate genes and the abundance of various immune cell types. **(A)** In AD, the ROSMAP database showed positive correlations between genes *SNN, LIMCH1*, and *MAPK10* with monocytes and eosinophils, while these genes were negatively correlated with B cells, natural killer (NK) cells, and CD4+ T cells. **(B)** The ADNI database revealed a positive correlation between *ERBB3, LIMCH1,* and *SACS* with NK-cells and CD8+ T-cells, while *CYCS, SLC25A18,* and *MAPK10* exhibited negative associations with these cell types. In GBM, **(D)** the TCGA dataset indicated that *ABAT, KCNJ10,* and *SLC25A18* were positively associated with monocytes and neutrophils, while they were negatively correlated with eosinophils. **(E)** The CGGA dataset demonstrated significant positive correlations for *ADIPOR2, SASH1*, and *KCNJ10* with CD8^+^ T-cells, monocytes, and eosinophils, and a strong negative correlation with B-cells. **(C,F)** Further categorization of these genes into mitochondrial epistasis and mitochondrial localization genes revealed distinct correlation patterns. **(C)** In AD, mitochondrial epistasis genes showed significant positive correlations with NK cells and neutrophils, while mitochondrial localization genes were most strongly associated with neutrophils. **(F)** In GBM, both types of genes displayed strong associations with eosinophils, CD4^+^ T cells, and CD8^+^ T cells, with the highest positive correlations observed for NK cells and CD4^+^ T cells. These findings provide valuable insights into the complex interplay between immune cells and the TME in AD and GBM, highlighting the potential role of mitochondrial genes in modulating immune responses within these diseases.

Immune cell interactions with the tumor microenvironment (TME) are crucial for influencing tumor development, metastasis, and treatment response. We analyzed methylation data from four datasets to determine the distribution of 24 candidate genes in seven immune cell types. In the ROSMAP dataset, significant positive correlations were observed between the expression of *SNN*, *LIMCH1*, and *MAPK10* with monocytes and eosinophils, while negative correlations were noted with B-cells, NK-cells, and CD4^+^ T-cells. In the ADNI dataset, *ERBB3*, *LIMCH1*, and *SACS* showed strong positive correlations with NK-cells and CD8^+^ T-cells, while *CYCS*, *SLC25A18*, and *MAPK10* were negatively correlated with NK-cells and CD8^+^ T-cells (Figures 9A,B).

In GBM, TCGA methylation data indicated that *ABAT*, *KCNJ10*, and *SLC25A18* had significant positive associations with monocytes and neutrophils, while negative associations were noted with eosinophils. In the CGGA dataset, *ADIPOR2*, *SASH1*, and *KCNJ10* demonstrated significant positive correlations with CD8^+^ T-cells, monocytes, and eosinophils, along with a strong negative correlation with B-cells (Figures 9D,E).

Categorizing these genes into mitochondrial epistasis and localization genes revealed distinct correlation patterns. In AD datasets, mitochondrial epistasis genes showed the strongest positive correlation with NK cells and neutrophils. Mitochondrial localization genes were most strongly associated with neutrophils, while significant positive correlations were also observed with NK cells, CD4^+^ T-cells (ROSMAP), and CD8^+^ T-cells (ADNI). In GBM datasets, a combination of mitochondrial epistasis and localization genes exhibited strong correlations with eosinophils, CD4+ T-cells, and CD8^+^ T-cells, with NK cells and CD4^+^ T-cells showing the most prominent positive associations (Figures 9C,F).

### 3.6 Survival analysis identifies cross-disease markers with differential prognostic impact in AD and GBM

**Figure 10:**
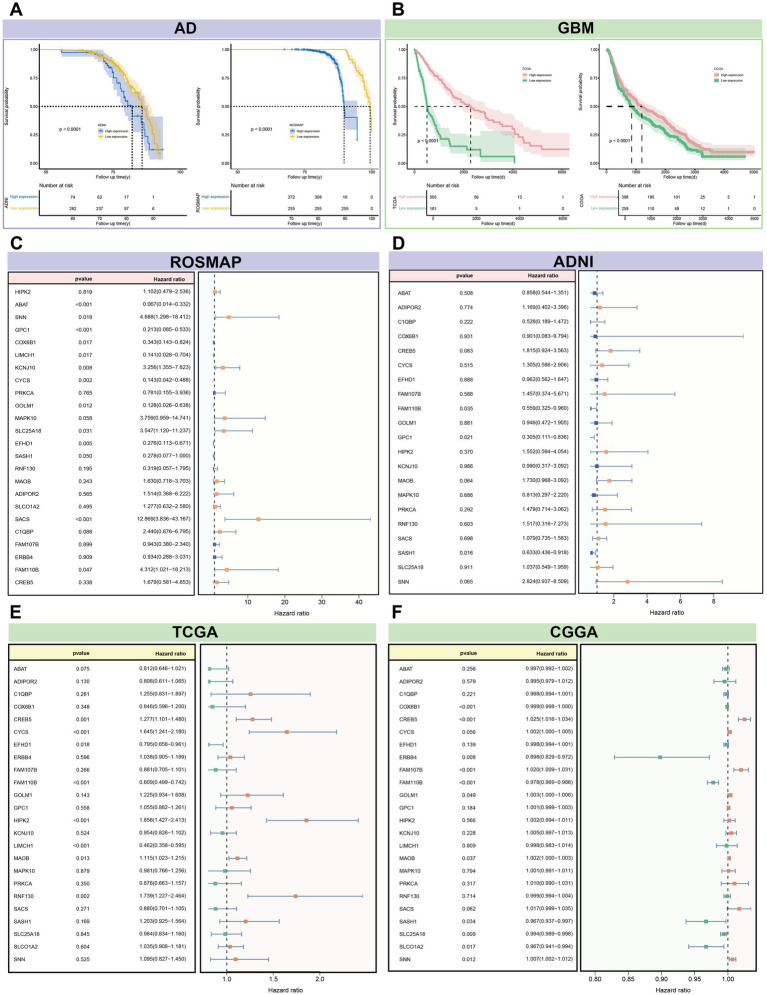
Survival analysis of 24 candidate genes associated with the risk of AD and GBM. **(A,B)** The survival curves for these genes revealed significant differentiation in disease risk, with a *p*-value threshold of less than 0.0001, indicating a profound impact on disease prognosis. The Cox regression analysis, accounting for multiple gene contributions, identified genes with significant associations (*p* < 0.05) in each dataset: **(C)** ROSMAP with 13 genes, **(D)** ADNI with 3 genes, **(E)** TCGA with 8 genes, and **(F)** CGGA with 11 genes. Notable genes with the most substantial impact on disease risk include LIMCH1 from the TCGA dataset for GBM (Hazard Ratio [HR] = 0.46, *p* = 2.27 × 10–9) and *SACS* from the ROSMAP dataset for AD (HR = 12.87, *p* = 3.51 × 10–5). Additionally, *GPC1* from the ADNI dataset showed a significant association with AD risk (HR = 0.30, *p* = 2.27 × 10–9), while *CREB5* from the CGGA dataset was notably associated with GBM risk (HR = 1.02, *p* = 6.91 × 10–8). The analysis also highlighted genes with opposing effects on disease risk between AD and GBM, such as *CYCS, FAM110B, GOLM1,* and *SLC25A18*. These genes may increase the risk of one disease while decreasing the risk of the other, suggesting distinct pathophysiological roles. Conversely, *EFHD1, LIMCH1, MAOB, COX6B1, SASH1*, and *SNN* demonstrated consistent contributions to risk across both diseases, indicating a shared molecular mechanism that could be targeted for therapeutic intervention. The integrated analysis underscores the complexity of gene expression patterns associated with AD and GBM risk and provides a foundation for further investigation into the molecular determinants of disease prognosis and treatment response.

Survival analysis of 24 candidate genes across four datasets revealed significant differences in AD and GBM risk (*p* < 0.0001). Multi-gene Cox regression analysis identified significant genes (*p* < 0.05) in each dataset: ROSMAP (13 genes), ADNI (3 genes), TCGA (8 genes), and CGGA (11 genes) (Supplementary Table S11). Key genes with the most pronounced effects on disease risk were identified for each dataset: in TCGA, *LIMCH1* (HR = 0.46, *p* = 2.27 × 10^−9^); in CGGA, *CREB5* (HR = 1.02, *p* = 6.91 × 10^−8^); in ROSMAP, *SACS* (HR = 12.87, *p* = 3.51 × 10^−5^), and in ADNI, *GPC1* (HR = 0.30, *p* = 2.27 × 10^−9^). Among these significant genes, *CYCS*, *FAM110B*, *GOLM1*, and *SLC25A18* demonstrated opposing effects on disease risk in AD and GBM, increasing risk in one while decreasing it in the other. In contrast, *EFHD1*, *LIMCH1*, *MAOB*, *COX6B1*, *SASH1*, and *SNN* consistently contributed to disease risk in both AD and GBM (Figure 10). Further screening of integrated gene expression data revealed four genes—*EFHD1*, *SASH1*, *FAM110B*, and *SLC25A18*—that were differentially expressed in both AD and GBM (Supplementary Figure S8).

### 3.7 Single-cell analysis reveals key gene associations with cell cycle and stemness in high-grade gliomas

**Figure 11:**
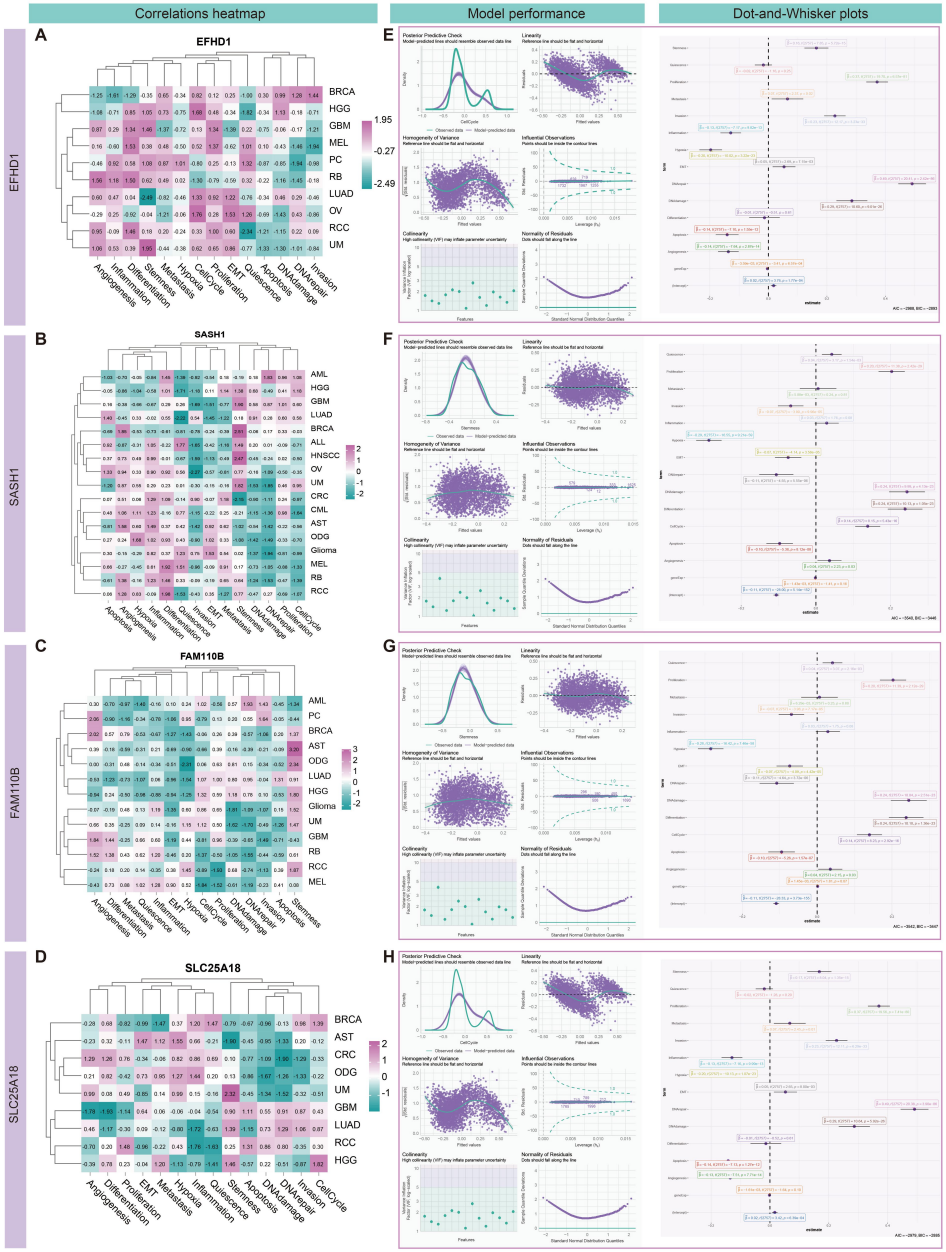
Correlation analysis and modeling of candidate genes with different cancer functional states in high-grade gliomas (HGG). **(A–D)** Heatmap showing the correlation between the normalized candidate genes and the different characteristics of different tumors. The analysis revealed significant positive correlations between the genes *EFHD1* and *SLC25A18* with cellular processes, particularly the cell cycle, with correlation coefficients (Cor) of 0.368 and 0.385, respectively (*P* < =0.001). Additionally, *SASH1* and *FAM110B* were found to be closely associated with tumor stemness, with Cor of 0.328 and 0.427, respectively (*P* < =0.001). **(E–H)** Generalized linear models were used to further evaluate these associations, with cell cycle and stemness as dependent variables and the remaining 14 gene characteristics as independent variables. This approach allowed us to explore the intricate relationships between gene expression and the complex biological processes in HGG. The modeling results demonstrated significant associations between *EFHD1* and *SLC25A18* with the cell cycle, with a determination coefficient (*R*^2^) of 0.79. Likewise, *SASH1* and *FAM110B* showed a substantial association with tumor stemness, with an *R*^2^ value of 0.522. These findings provide valuable insights into the molecular interplay within HGG and underscore the potential of these candidate genes as indicators of the disease’s functional states.

Single-cell level analysis using the CancerSEA database (Yuan et al., 2019) revealed significant correlations between four candidate genes and functional states of high-grade gliomas (HGG) in the central nervous system (CNS). Notably, *EFHD1* and *SLC25A18* exhibited strong positive correlations with cellular processes related to the cell cycle (Cor = 0.368/0.385, *P* < =0.001), whereas *SASH1* and *FAM110B* were found to be closely associated with tumor stemness (Cor =0.328/0.427, *P* < =0.001) (Figures 11A–D and Supplementary Table S12).

To further validate these associations, we employed generalized linear models that incorporated a comprehensive set of 14 gene characteristics in HGG. These characteristics spanned a range of biological processes, including gene expression, angiogenesis, apoptosis, cell cycle, differentiation, DNA damage, DNA repair, epithelial-mesenchymal transition (EMT), hypoxia, inflammation, invasion, metastasis, proliferation, quiescence, and stemness. In these models, cell cycle and stemness were set as the dependent variables, while the remaining characteristics served as independent variables, allowing us to capture the complex interplay between gene expression and HGG biology. Our models demonstrated that *EFHD1* and *SLC25A18* were significantly associated with the cell cycle (*R*^2^ = 0.790), while *SASH1* and *FAM110B* showed a notable association with tumor stemness (*R*^2^ = 0.522) (Figures 11E–H and Supplementary Tables S13–S16).

### 3.8 Pseudotime analysis reveals distinct cellular lineages and dynamic expression patterns of key genes in AD and GBM progression

We conducted pseudotime analysis on sn/scRNA-seq datasets from AD and GBM to explore dynamic cellular features. The heatmap illustrated three distinct lineages and six feature clusters within each dataset, with each cluster associated with unique biological functions. For instance, in AD, Cluster 1 (C1) was enriched for immune processes, including complement activation and cell adhesion. Cluster 2 (C2) was involved in the regulation of the MAPK cascade, while Cluster 4 (C4) related to intermediate filaments and the cytoskeleton. In GBM, Cluster 1 (C1) showed a response to essential metal ions such as copper and zinc and was associated with synaptic potentiation. Cluster 2 (C2) correlated with the blood–brain barrier, and Cluster 3 (C3) was linked to membrane proteins and lipolytic processes (Supplementary Figure S9).

tSNE maps further elucidated the potential evolutionary paths of various cell types in AD and GBM. In AD, the expression levels of Astrocytes, Oligodendrocytes, and OPCs were notably higher in the later stages across the three spectral time series. Specifically, *EFHD1* exhibited increased expression at the late pseudotime stage of Lineage 1. *SASH1* showed elevated expression at the late pseudotime stages across Lineages 1, 2, and 3. The expression of *FAM110B* fluctuated similarly across different lineages and time periods, while *SLC25A18* displayed increased expression at the late pseudotime stage of Lineage 3.

In GBM, *EFHD1* expression was heightened in oligodendrocytes and tumor cells during the middle and late pseudotime stages of Lineage 1. *SASH1* demonstrated a decreasing expression trend from pre-pseudotime tumor cells and myeloid cells. *FAM110B* expression was upregulated in tumor cells and some oligodendrocytes during the middle and late stages of Lineages 1 and 3. *SLC25A18* expression was elevated in tumor cells and a subset of astrocytes and oligodendrocytes at the late pseudotime stages of Lineages 1 and 3.

### 3.9 Single-cell co-expression network and gene enrichment analyses reveal cell type-specific modules and mitochondrial gene associations in AD and GBM

**Figure 12:**
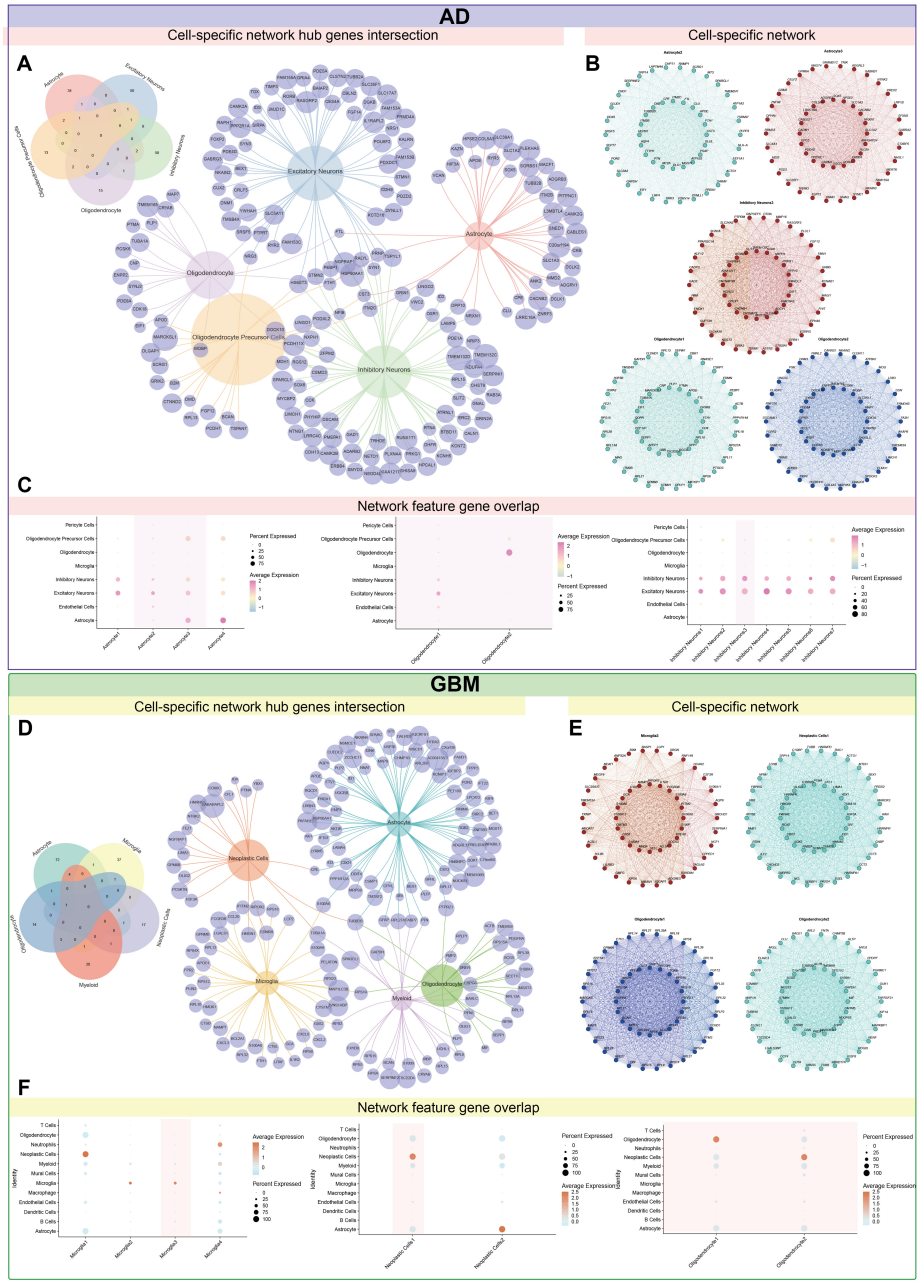
Identification of the crucial modules related to candidate genes by hdWGCNA. Gene co-expression networks were established at the single-cell level for both diseases, revealing the interaction patterns among the candidate genes. The analysis provides a comprehensive view of the molecular crosstalk within cell types associated with these diseases. By aggregating hub genes from each cell’s modules, their prevalence across cell types was quantified. **(A–C)** The most recurrent hub genes identified were *FTH1,* which was prevalent in Astrocytes, Oligodendrocytes, and OPCs. *HS6ST3* was found in excitatory neurons, inhibitory neurons, and oligodendrocyte precursor cells. These genes are considered central in the gene co-expression network, indicating their potential role in key cellular processes. **(D–F)** In GBM, the gene *TUBB2B* emerged as the most frequently occurring hub gene, present in Astrocytes, Neoplastic Cells, Myeloid cells, and Oligodendrocytes. This highlights its potential as a central player in the disease’s cellular dynamics. Among the identified hub genes, a subset was implicated in mitochondrial function: (1) Mitochondrial epistasis genes, including *HS6ST3, DOCK10, DPP10, NRG3*, and *NRXN1*, are suggested to regulate mitochondrial activity, which is critical for cellular energy metabolism and other functions. (2) Mitochondrial localization genes, *HSP90AA1* and *PRNP*, are involved in the precise targeting and localization of proteins within the mitochondria, essential for maintaining cellular health. These findings provide valuable insights into the molecular mechanisms underlying the cellular heterogeneity in AD and GBM. The identification of crucial hub genes and their involvement in mitochondrial functions suggests their potential as central players in the pathogenesis of these diseases. These insights may contribute to the development of targeted therapeutic strategies.

We identified cell types in AD and GBM using candidate differential genes from multi-omics data analysis. The identified AD cell types include astrocytes, oligodendrocytes, oligodendrocyte precursor cells (OPCs), and excitatory/inhibitory neurons (Supplementary Tables S17–S21). In GBM, the identified cell types are astrocytes, microglia, oligodendrocytes, myeloid cells, and neoplastic cells (Supplementary Tables S22–S26). To investigate potential gene interactions, we constructed single-cell gene co-expression networks for AD and GBM. In AD, the co-expression network revealed modules predominantly involving astrocytes (4 modules), inhibitory neurons (7 modules), and oligodendrocytes (2 modules) (Supplementary Figure S10). In GBM, co-expression modules were detected in microglia (4 modules), neoplastic cells (2 modules), and oligodendrocytes (2 modules) (Supplementary Figure S12). We identified hub genes from each cell module and assessed their recurrence across cell types. The most recurrent hub genes in AD were *FTH1* (identified in astrocytes, oligodendrocytes, and OPCs) and *HS6ST3* (found in excitatory neurons, inhibitory neurons, and OPCs). In GBM, *TUBB2B* emerged as the most frequently identified hub gene, observed in astrocytes, neoplastic cells, myeloid cells, and oligodendrocytes. Among the identified hub genes, mitochondrial epistasis-related genes included *HS6ST3*, *DOCK10*, *DPP10*, *NRG3*, and *NRXN1*. Meanwhile, genes with mitochondrial localization included *HSP90AA1* and *PRNP*. Figure 12 and Supplementary Figures S11, S13 illustrated the overlap between sub-modules within individual cell types and differentiation genes, correlations between modules, as well as the network and distribution of hub genes across cellular subsets. Notably, oligodendrocytes demonstrated the most pronounced variation in module distribution, suggesting distinct functional roles in both AD and GBM pathologies.

Gene enrichment analyses further revealed specific associations. In AD, *SASH1* was associated with modules involving astrocytes (Astrocytes3), inhibitory neurons (Inhibitory Neurons3), and oligodendrocytes (Oligodendrocytes2). *FAM110B* was linked to astrocytes and inhibitory neurons (Inhibitory Neurons3), while *EFHD1* showed enrichment primarily in oligodendrocytes (Oligodendrocytes1). The Astrocyte2 module was found to have strong associations with excitatory neurons and exhibited functional enrichment for the glutamate catabolic process (GO:0006538) and intracellular iron ion sequestration (GO:0006880). Notably, gene expression synergism was observed between excitatory and inhibitory neurons, indicating potential interactions affecting neuronal signaling. Inhibitory Neurons3 were particularly involved in retinal ganglion cell axon guidance (GO:0031290) and the glutamate receptor signaling pathway (GO:0007215). Oligodendrocyte modules (Oligodendrocyte1 and Oligodendrocyte2) were enriched for endoplasmic reticulum proteins (GO:0045047) and processes regulating basement membrane assembly during embryoid body morphogenesis (GO:1904261), respectively (Supplementary Figure S11).

In GBM, gene enrichment analyses also identified *SASH1* associated with microglia (Microglia3) and oligodendrocytes (Oligodendrocytes1). *FAM110B* was linked to neoplastic cells (Neoplastic Cells1) and oligodendrocytes (Oligodendrocytes2). The Microglia3 module was involved in processes such as leukocyte aggregation (GO:0070486), adiponectin regulation (GO:0070163), astrocyte differentiation (GO:0048708), and excitatory postsynaptic potential (GO:0090394). Oligodendrocyte modules (Oligodendrocyte1 and Oligodendrocyte2) were associated with the regulation of protein depolymerization (GO:1901879) and protein targeting to the endoplasmic reticulum (GO:0045047), respectively (Supplementary Figure S13).

## 4 Discussion

Emerging evidence suggests both positive and inverse relationships between AD and GBM risk (Zabłocka et al., 2021; Cai et al., 2022). AD is characterized by neurodegeneration, while GBM involves uncontrolled cell proliferation. Despite their differences, both share mitochondrial dysfunction as a common pathological feature. In AD, mitochondrial impairment reduces energy production and increases oxidative stress, leading to neuronal death (Ebanks et al., 2020). Conversely, in GBM, mitochondrial abnormalities support tumor growth and resistance to apoptosis, promoting malignancy (Wang S. et al., 2024). These opposing effects underscore the complex role of mitochondria in disease mechanisms. Multi-omics approaches and machine learning algorithms are pivotal in identifying key genes and pathways involved in AD and GBM.

First, we explored the characterization of different cell types and their marker genes in AD and GBM based on single-cell data. Oligodendrocytes in both diseases were enriched for mitochondria-associated functions, including the mitochondrial morphogenetic pathway in AD and ATP synthesis coupled with electron transport in GBM. In AD, *LPAR1*, a key mitochondrial epistasis gene, influences mitochondrial dynamics, energy metabolism, and apoptosis, contributing to neuroinflammation and neuronal survival. In GBM, *LPAR1* promotes tumor growth and invasion through altered mitochondrial bioenergetics (Choi et al., 2010). These findings highlight *LPAR1*’s potential as a therapeutic target in both AD and GBM. Additionally, GBM Neoplastic cells, Astrocytes, and Oligodendrocytes exhibited enrichment in NADH dehydrogenase complex (Complex I) genes. Mutations in *MT-ND1*, *MT-ND2*, *MT-ND4*, and *MT-ND5*, crucial for Complex I function, lead to altered bioenergetics and increased oxidative stress, contributing to GBM’s aggressiveness and therapeutic resistance (Viale et al., 2015). In comparing AD and GBM, mitochondria play divergent roles: sustaining neuronal signaling in AD and meeting high-energy demands in GBM. Oligodendrocyte function, linked to myelin production, may be compromised in AD, impacting axonal signaling, whereas in GBM, it may be adapted to support the tumor microenvironment. Understanding these roles could reveal shared or distinct regulatory mechanisms and offer new avenues for intervention.

Cellular communication analysis emphasized the importance of the microenvironment in AD and GBM, particularly the regulatory roles of astrocytes and oligodendrocytes. Astrocytes interact with GBM cells to promote tumor growth, invasion, and therapy resistance, partly through extracellular vesicles and mitochondrial transfer mediated by GAP43 (Brandao et al., 2019; Zhang et al., 2020; Watson et al., 2023). Astrocytes also engage in complex interactions with oligodendrocytes, crucial for glial development, disease progression, and tissue regeneration (Linnerbauer et al., 2020; Hu et al., 2023). These interactions, which occur via direct contact and secreted factors, significantly impact CNS homeostasis and pathogenesis. Understanding the cross-talk between astrocytes and microglia is also essential, as it contributes to GBM progression through cytokines and chemokines (Fan et al., 2024). Insights into these cellular relationships could lead to new therapeutic strategies for GBM and other CNS diseases.

Our findings revealed significant enrichment of the *APP* (amyloid precursor protein) signaling pathway in both AD and GBM. In AD, *APP* processing generates Aβ, leading to plaque formation, neuronal damage, and inflammation, ultimately contributing to cognitive decline (Hardy and Selkoe, 2002). In GBM, APP has been implicated in promoting cell proliferation and invasion (Lee et al., 2021). Mitochondria are central to these processes: in AD, Aβ interacts with mitochondria, causing dysfunction and oxidative stress, exacerbating neuronal injury (Atamna and Frey, 2007); in GBM, mitochondria regulate energy metabolism, apoptosis, and tumor cell invasiveness (Wu et al., 2022). Additionally, the shared involvement of the NRXN pathway in both diseases highlights its potential role. Neurexins, such as *Neurexin-1β*, stimulate the PI3K pathway in glioma cells, promoting growth (Venkatesh et al., 2015; Yun et al., 2023), and are implicated in synaptic dysfunction in AD (Zhang R. et al., 2023). This connection between *NRXN1*, mitochondrial pathways, and neurological function presents an avenue for understanding and treating these disorders (Nakamura and Kennedy, 2021; Levy et al., 2022).

Differential enrichment analysis revealed significant gene expression patterns in AD and GBM. Upregulated genes in both diseases were enriched in pathways related to mitosis, osteoblast morphogenesis, and transcriptional maintenance, indicating shared mechanisms of cell proliferation and structural remodeling (De La Torre, 2002; Dobson et al., 2020; Perluigi et al., 2024). Mitochondria are crucial in these processes, regulating energy production, oxidative stress, and apoptosis. Mitochondrial dysfunction in both AD and GBM has been linked to increased cell proliferation and altered differentiation. In GBM, these abnormalities increase ROS production, leading to DNA damage and tumorigenesis (Kim et al., 2006), while in AD, they exacerbate neuronal damage and synaptic loss (Chen W. et al., 2023). Downregulated genes in both diseases were linked to nuclear transcription, synaptic signaling, and calcium ion transport. Mitochondrial dysfunction affects calcium homeostasis, impacting neuronal function in AD and tumor cell viability in GBM (Chen W. et al., 2023; An et al., 2024; Kazanietz and Cooke, 2024; Lin H. et al., 2024). These insights highlight the shared and distinct mechanisms underlying AD and GBM and suggest potential therapeutic targets.

Combining single-cell signature genes and machine learning algorithms, we identified 24 cell-specific mitochondria-associated markers, which can be categorized into two groups based on their relationship with mitochondria: mitochondria epitope-associated (MT-Inter) and mitochondria-located genes (MT-Locate). The associations of these marker genes with immune cells were explored using methylation data. In AD, both classes of genes showed strong associations with neutrophil levels. Neutrophils migrate into the brain parenchyma and adhere to blood vessels in AD, releasing extracellular traps (NETs) that exacerbate neuroinflammation and tissue damage (Pietronigro et al., 2017; Dong et al., 2018; Aries and Hensley-McBain, 2023). Mitochondria in neutrophils contribute to their development, chemotaxis, effector functions, and cell death, playing an essential role in disease progression (Dong et al., 2018; Peng et al., 2023). In the tumor microenvironment, neutrophils adapt to glucose limitation by utilizing oxidative mitochondrial metabolism, particularly fatty acid oxidation, to sustain reactive oxygen species (ROS) production and immune suppression (Rice et al., 2018). These findings underscore the crucial role of mitochondria in neutrophil biology and their potential as therapeutic targets.

In GBM, we found associations between the two gene classes and immune cells, including eosinophils, CD4+ T-cells, and CD8+ T-cells. Eosinophils, though traditionally linked to allergic responses, modulate the GBM tumor microenvironment, with increased eosinophil-associated cytokine levels correlating with better survival and enhanced T-cell infiltration (Herold-Mende et al., 2023). Mitochondria are essential for eosinophil apoptosis and survival, modulated by glucocorticoids and nitric oxide (Ilmarinen et al., 2014). Mitochondrial dysfunction in CD8+ T-cells impairs their anti-tumor activity, affecting energy metabolism and increasing apoptosis (Yang et al., 2010; Zhang et al., 2022). Interestingly, a combination of high CD4+ and low CD8+ tumor-infiltrating lymphocytes (TILs) predicts poor prognosis in GBM, indicating a complex relationship between T-cell subtypes and outcomes (Waziri et al., 2008; Abarca-Rojano et al., 2009; Han et al., 2014; Lee et al., 2015; Surace et al., 2021; Du et al., 2023; Kim et al., 2023). Understanding these immune interactions and mitochondrial dynamics offers promising therapeutic targets.

From survival and differential analyses, we identified four key mitochondria-related genes: *EFHD1*, *SASH1*, *FAM110B*, and *SLC25A18*, which play critical roles in mitochondrial function, cellular metabolism, and signaling pathways. *EFHD1*, a mitochondrial calcium-binding protein, regulates calcium homeostasis and suppresses tumor metastasis via the Hippo/YAP pathway (Mun et al., 2021; Meng et al., 2023). It also influences calcium-dependent transcriptional co-activators or repressors, modulating *ESR1* activity in the *Transcriptional Regulation by the AP-2 (TFAP2) Family of Transcription Factors (R-HSA-8864260.2)* pathway (Nassa et al., 2019). This interaction integrates TFAP2-and *EFHD1*-mediated signals to regulate hormone-related genes such as VEGFA, contributing to cellular growth and angiogenesis. Besides, *EFHD2* is implicated in neurological disorders, including AD, and regulates macrophage function in GBM, presenting potential for immunotherapy (Vega, 2016; Zhang et al., 2023a). *SASH1*, acting as a tumor suppressor and mitochondrial epistasis gene, regulates oxidative stress, cell adhesion, and migration (Martini et al., 2011; Yang et al., 2012, 2015; Cazzaro et al., 2023; Lundberg et al., 2023). It interacts with *SFN* (14–3-3σ) in the *TP53 Regulates Transcription of Cell Cycle Genes (R-HSA-6791312.5)* pathway, enhancing *TP53*-mediated responses to DNA damage by stabilizing CDK inhibitors and enforcing G2/M checkpoint arrest. This interaction contributes to genomic stability by coordinating cell cycle arrest, DNA repair, and apoptosis pathways (Segal et al., 2023). *FAM110B* is associated with cell proliferation and cytoskeletal organization (Li et al., 2024; Yan et al., 2020). In the *Deregulated CDK5 Triggers Multiple Neurodegenerative Pathways in Alzheimer’s Disease (R-HSA-8862803.4)* pathway, it interacts with *YWHAE* (14–3-3ε), stabilizing the cytoskeleton and modulating stress responses (Cho et al., 2022). Together, they mitigate CDK5-induced neurodegeneration by regulating Tau hyperphosphorylation, oxidative stress, and neuronal apoptosis, making *FAM110B* and *YWHAE* potential therapeutic targets in Alzheimer’s disease. *SLC25A18* is a mitochondrial carrier protein involved in glutamate transport, linking amino acid metabolism to the TCA cycle by importing L-glutamate into the mitochondrial matrix, where it is converted to 2-oxoglutarate (Yan et al., 2020; Wang et al., 2021; Wang S. et al., 2024; Watanabe et al., 2021; Taylor et al., 2022; Ren et al., 2023). In the *Malate–Aspartate Shuttle (R-HSA-9856872.1)* pathway, *SLC25A18* supports energy production, biosynthesis, and mitochondrial homeostasis, integrating metabolite transport with oxidative phosphorylation to ensure metabolic flexibility and energy balance. These genes are potential targets for therapeutic interventions in both neurodegeneration and cancer. The detailed pathway regulatory relationships are shown in Supplementary Figure S14.

Lineage analysis showed distinct expression patterns for these genes. In AD, *EFHD1*, *SASH1*, and *SLC25A18* were significantly elevated in oligodendrocytes, OPCs, and astrocytes at late pseudotemporal stages. In GBM, these genes were associated with tumor cells and oligodendrocytes at different stages. Oligodendrocytes, critical for CNS myelination, depend on mitochondrial function for energy production and lipid synthesis. Dysfunction in these cells, influenced by factors such as *β*-amyloid and neurofibrillary tangles, contributes to neuroinflammation, oxidative stress, and apoptosis in AD, whereas in GBM, oligodendrocytes enhance tumor cell migration and therapy resistance (Cai and Xiao, 2016; Rinholm et al., 2016; Butt et al., 2019; Kawashima et al., 2019; Meyer and Rinholm, 2021; Chen P. et al., 2023; Hide and Komohara, 2020). Targeting oligodendrocytes and OPCs may offer therapeutic opportunities in both AD and GBM.

The identification of mitochondrial disease-specific markers (*EFHD1*, *SASH1*, *FAM110B*, and *SLC25A18*) highlights shared mechanisms, such as oxidative phosphorylation, calcium signaling, and immune responses, that can serve as therapeutic targets in both AD and GBM. These markers enable patient stratification based on mitochondrial dysfunction, paving the way for personalized treatments addressing neurodegenerative and oncogenic processes. For instance, modulating oxidative stress and mitochondrial metabolism could simultaneously enhance neuronal survival in AD and suppress metabolic adaptation in GBM. Additionally, combination therapies targeting shared pathways, such as calcium homeostasis and energy metabolism, offer the potential for synergistic effects. These findings underscore the translational value of mitochondrial biomarkers and the need for further experimental validation to refine their therapeutic application.

The classification of GBM based on IDH mutation status is critical for both diagnosis and treatment planning, as patients with IDH-mutant GBM generally exhibit a significantly better prognosis compared to those with IDH wild-type tumors (Han et al., 2020). In our study, we identified four key marker genes (*EFHD1, SASH1, FAM110B*, and *SLC25A18*) and evaluated their differential expression across IDH subtypes using the CGGA dataset. The results revealed significant expression differences between IDH-mutant and IDH-wildtype GBM, highlighting their potential as biomarkers for stratifying gliomas by IDH status and severity. Among the markers, *FAM110B* displayed the most pronounced differential expression. It was significantly overexpressed in IDH-mutant gliomas across all grades of severity, suggesting its strong association with the IDH-mutant phenotype. This consistent overexpression underscores its potential role in the molecular mechanisms driving the relatively favorable prognosis of IDH-mutant gliomas. *SLC25A18*, another key marker, showed the highest expression levels in lower-grade gliomas (WHO grade II) with IDH mutation and in IDH-mutant, 1p19q co-deleted (LGG) subtypes. This expression pattern aligns with the less aggressive clinical behavior typically observed in these glioma subtypes and highlights *SLC25A18*’s potential involvement in the metabolic adaptations associated with IDH mutations (Supplementary Figure S15). These findings emphasize the relevance of IDH status in interpreting the functional roles of these markers and their correlation with glioma severity. Further functional studies are warranted to elucidate how these markers contribute to the distinct biological pathways and clinical outcomes observed in IDH-mutant gliomas. Additionally, their differential expression profiles may provide insights into IDH-specific therapeutic targets, offering opportunities for more personalized approaches in glioma treatment.

To elucidate gene interactions, we established a single-cell co-expression network and identified *FTH1*, *HS6ST3*, and *TUBB2B* as hub genes across multiple cell types. *HS6ST3* is involved in the biosynthesis of heparan sulfate (HS), a glycosaminoglycan that regulates mitochondrial function under stress conditions (Lehri-Boufala et al., 2015). This suggests an indirect link between *HS6ST3* and mitochondrial processes, and it has been identified as a hub gene in the GBM protein–protein interaction network (Xie et al., 2018). *HS6ST3* may impact mitochondrial regulation in both AD and GBM (El Hayek et al., 2023). *TUBB2B*, encoding tubulin beta 2B, is linked to cortical malformations and microtubule dysfunction, common in neurodegenerative diseases like AD (Sferra et al., 2020; Soliman et al., 2022). Although associated with AD pathology, *TUBB2B* has no direct evidence linking it to GBM, requiring further investigation.

**Figure 13:**
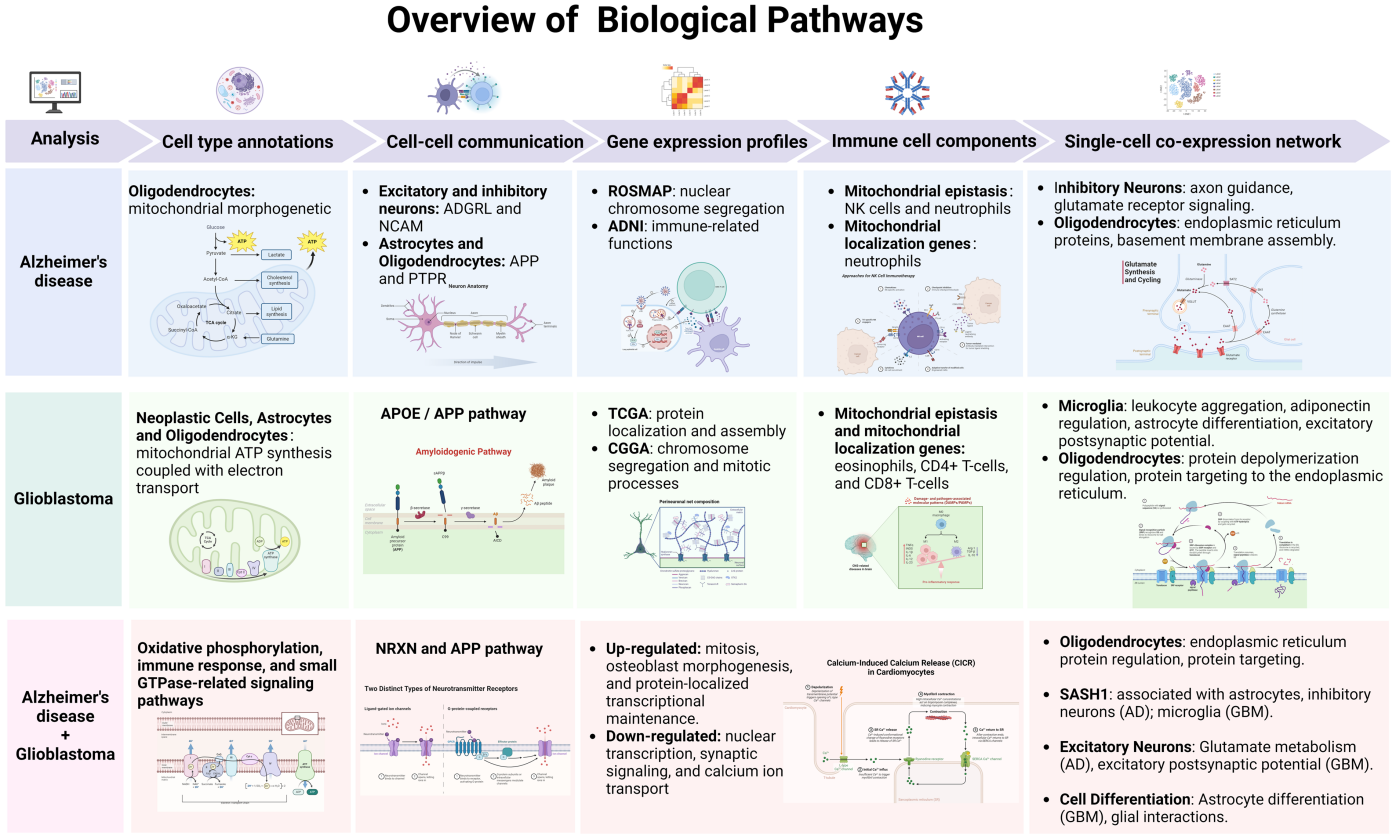
Overview of disease-specific and shared biological pathways in Alzheimer’s disease and glioblastoma. This figure summarizes the key biological pathways identified through our analytical pipeline for AD, GBM, and their shared mechanisms. The pathways are categorized across five analytical dimensions: cell type annotations, cell–cell communication, gene expression profiles, immune cell components, and single-cell co-expression networks. In AD, pathways include mitochondrial morphogenesis in oligodendrocytes, APP/NCAM signaling in astrocytes and inhibitory neurons, and immune-related nuclear functions. In GBM, critical processes involve mitochondrial ATP synthesis in neoplastic cells, astrocytes, and oligodendrocytes, as well as mitotic and protein localization pathways. Shared pathways encompass oxidative phosphorylation, calcium signaling, and immune responses, with notable roles for *NRXN, APP*, and small GTPase signaling. The figure highlights disease-specific and overlapping mechanisms, providing a comprehensive overview of the biological context for potential therapeutic targets.

Co-expression module analysis revealed significant associations of hub genes with oligodendrocytes, followed by astrocytes and inhibitory neurons, indicating contributions to both diseases. Astrocytes are crucial in AD and GBM; in AD, they contribute to neuroinflammation, oxidative stress, and Aβ clearance (Bi et al., 2022), while in GBM, they support tumor growth and invasion, making them potential therapeutic targets (Brandao et al., 2019). Live imaging has shown limited mitochondrial dynamics in mature astrocytes *in vivo* compared to cultured cells, suggesting tightly regulated mitochondrial activity (Bergami and Motori, 2020). Astrocyte-oligodendrocyte interactions are critical for CNS remyelination, blood–brain barrier (BBB) integrity, and synaptogenesis (Hu et al., 2023; Molina-Gonzalez et al., 2023) Understanding these interactions could inform therapeutic strategies for CNS diseases. The AD-, GBM-specific and common biological pathways identified in our pipeline are summarized in Figure 13.

Previous studies have demonstrated that mitochondrial dysfunction in Parkinson’s disease (PD), Huntington’s disease (HD), and amyotrophic lateral sclerosis (ALS) primarily affects pathways related to oxidative phosphorylation, mitochondrial dynamics, and calcium signaling (Green and Reed, 1998; Henrich et al., 2023). For example, *MT-ND1, MT-ND2, MT-ND4,* and *MT-ND5*, which are components of the mitochondrial electron transport chain, have been shown to be downregulated in both PD and ALS, reflecting impaired mitochondrial energy metabolism (Johri and Beal, 2012). In contrast, our study on AD and GBM highlights a distinct enrichment of mitochondrial pathways related to energy metabolism and ATP synthesis, particularly in GBM, where mitochondrial dysfunction is associated with tumor progression and cell survival. In AD, we observed significant enrichment in pathways linked to synaptic transmission and neuronal signaling, which are not as prominently affected in PD or ALS (Kori et al., 2016). Moreover, immune-related mitochondrial pathways involving microglia activation were shared across AD and GBM, which is consistent with findings in ALS and HD, where neuroinflammation plays a central role in disease pathogenesis (Chen K. et al., 2023). However, the specific involvement of *APP, TREM2*, and *CD74* in microglial activation was particularly pronounced in AD and GBM, suggesting a more disease-specific activation of the immune system in these two conditions. In contrast, PD and ALS exhibit different immune activation profiles, with a more prominent role of astrocytes and other glial cells (Philips and Rothstein, 2014).

While our study highlights potential mitochondrial links between AD and GBM, several limitations must be acknowledged. The current findings, based on bioinformatics analyses of multi-omics datasets, lack experimental validation and precise mechanistic insights. Future research should integrate genomic, proteomic, and metabolomic studies, alongside cell-based and in vivo models, to validate the roles of key markers (*EFHD1, SASH1, FAM110B*, and *SLC25A18*) in mitochondrial dysfunction. Additionally, further investigation into the differential roles of these genes across IDH subtypes—such as *FAM110B* in IDH-mutant gliomas—and their therapeutic potential is needed. The interaction between clinical factors (e.g., IDH status and MGMT methylation) and our prognostic model also requires clarification through analysis of annotated clinical samples. Validation in larger, multi-ethnic cohorts and longitudinal datasets tracking mitochondrial changes across disease stages will enhance generalizability and identify critical therapeutic windows. Addressing these limitations will advance our understanding of mitochondrial dysfunction in AD and GBM, guiding the development of targeted therapies.

## 5 Conclusion

In summary, our research leverages single-cell data and applies a suite of 10 machine learning algorithms to discern mitochondria-related cell-specific markers, thereby contributing to the field’s understanding of AD and GBM. By integrating gene expression and methylation data, we have meticulously validated these markers and charted the expression patterns of mitochondrial features common to both diseases across various cell types. The identification of *EFHD1, SASH1, FAM110B*, and *SLC25A18* as significant cross-disease markers sheds light on the shared and divergent mitochondrial mechanisms at play in AD and GBM. Our findings suggest a potential role for oligodendrocytes and their interactions with astrocytes in the pathogenesis of both diseases, with a particular focus on the APP signaling pathway. This insight provides a fresh perspective on the cellular dynamics that may underlie these conditions. Furthermore, the discovery of key hub genes, including *HS6ST3* and *TUBB2B*, within cellular subpopulations, and their association with a cell-specific co-expression network associated with these mitochondrial markers, suggests a complex interplay of genetic factors that could influence disease progression and response to treatment. Our work strives to add complexity to the mitochondrial narrative in AD and GBM, with the ultimate aim of enhancing clinical relevance and therapeutic potential.

## Data Availability

The software used in this study is publicly available. Libraries were used according to their vignettes with parameter descriptions and versions as specified in the Methods. Custom code is available at GitHub (https://github.com/xxulab1230/AD_GBM).

## Footnotes

^1^ https://www.cancer.gov/tcga

^2^ http://www.cgga.org.cn/

^3^ https://community.10xgenomics.com/t5/Customer-Developed-Protocols/ct-p/customer-protocols

^4^ https://mlr-org.com/
