# Supplementary material for: Deciphering novel mitochondrial signatures: multi-omics analysis uncovers cross-disease markers and oligodendrocyte pathways in Alzheimer’s disease and glioblastoma
Source: Front Aging Neurosci. 2025 Feb 13;17:1536142. doi: 10.3389/fnagi.2025.1536142 (PMC11865232; doi:10.3389/fnagi.2025.1536142)
Supplement: Supplementary file 1 [file Data_Sheet_1.docx]

**Extended Data Figures**

Deciphering novel mitochondrial signatures: multi-omics analysis uncovers cross-disease markers and oligodendrocyte pathways in Alzheimer's disease and Glioblastoma

[Extended Data Fig.1 Extended data for cell communication analysis of AD single-cell data. 2](#_Toc188576628)

[Extended Data Fig.2 Extended data for cell communication analysis of GBM single-cell data. 4](#_Toc188576629)

[Extended Data Fig.3 Extended data for transcriptomic characterization of AD and GBM. 6](#_Toc188576630)

[Extended Data Fig.4 Overlapping patterns of gene enrichment observed in AD and GBM. 8](#_Toc188576631)

[Extended Data Fig.5 Integrative analysis reveals key mitochondrial markers and their intersections in AD and GBM. 10](#_Toc188576632)

[Extended Data Fig.6 The specific expression patterns of the identified candidate marker genes in various cell types of AD. 12](#_Toc188576633)

[Extended Data Fig.7 The specific expression patterns of the identified candidate marker genes in various cell types of GBM. 13](#_Toc188576634)

[Extended Data Fig.8 Expression patterns for four candidate genes in four independent datasets. 14](#_Toc188576635)

[Extended Data Fig.9 Lineage-specific gene expression dynamics in AD and GBM. 16](#_Toc188576636)

[Extended Data Fig.10 Construction of hdWGCNA network for AD. 19](#_Toc188576637)

[Extended Data Fig.11 Detailed analysis of the co-expression modules of key cell types in AD. 21](#_Toc188576638)

[Extended Data Fig.12 Construction of hdWGCNA network for GBM. 23](#_Toc188576639)

[Extended Data Fig.13 Detailed analysis of the co-expression modules of key cell types in GBM. 25](#_Toc188576640)

[Extended Data Fig.14 Major pathways involving *EFHD1, SASH1, FAM110B*, and *SLC25A18.* 27](#_Toc188576641)

[Extended Data Fig.15 Differential expression of four key marker genes across IDH subtypes and Glioma severity grades*.* 30](#_Toc188576642)


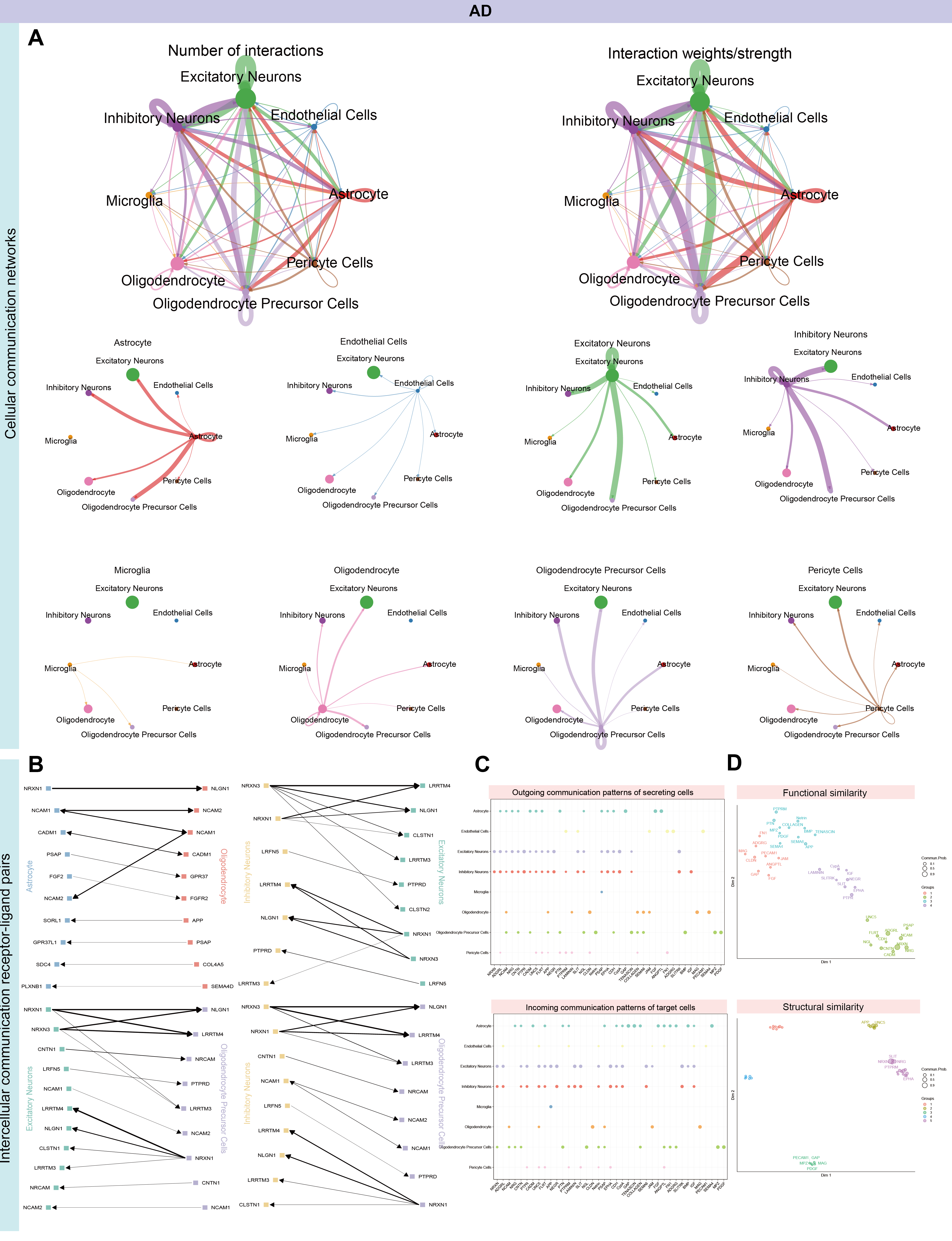


# Extended Data Fig.1 Extended data for cell communication analysis of AD single-cell data.

**(A)** The cell-cell communication network aggregated the interactions among key cell types in the brain, revealing a complex landscape of signaling. Notable receptor-ligand interactions were identified, particularly between Oligodendrocyte Precursor Cells (OPCs) and Astrocytes, as well as between Excitatory and Inhibitory Neurons. These interactions suggested a critical role for these cell types in neuronal communication, potentially influencing disease progression in AD. **(B-C)** The visualization of cell-cell communication, mediated by specific ligand-receptor pairs, underscored the significance of NRG3-ERBB4, NRXN3-LRRTM4, and NRXN1-NLGN1 in Astrocytes, Excitatory/Inhibitory Neurons, and OPCs. The categorization of signals into efferent and afferent patterns, along with the identification of five efferent and four afferent patterns, provided insight into the directional flow of signals within the neural network. This directional specificity may be crucial for understanding the coordinated responses of neural cells to various stimuli. **(D)** The manifold and classification learning analysis of signaling networks offered a deeper understanding of the relationships between different signaling pathways. By quantifying their similarities, we can discern groups of pathways that may share functional or structural roles within the cellular communication network. High functional similarity indicates that major senders and receivers are analogous, suggesting that these pathways or ligand-receptor pairs could have overlapped or complementary functions. Structural similarity compared the overall network architecture, highlighting pathways that may operate through similar mechanisms despite differences in their specific components. This analysis provided a framework for further investigation into the functional redundancy and network robustness in the context of neurological diseases.


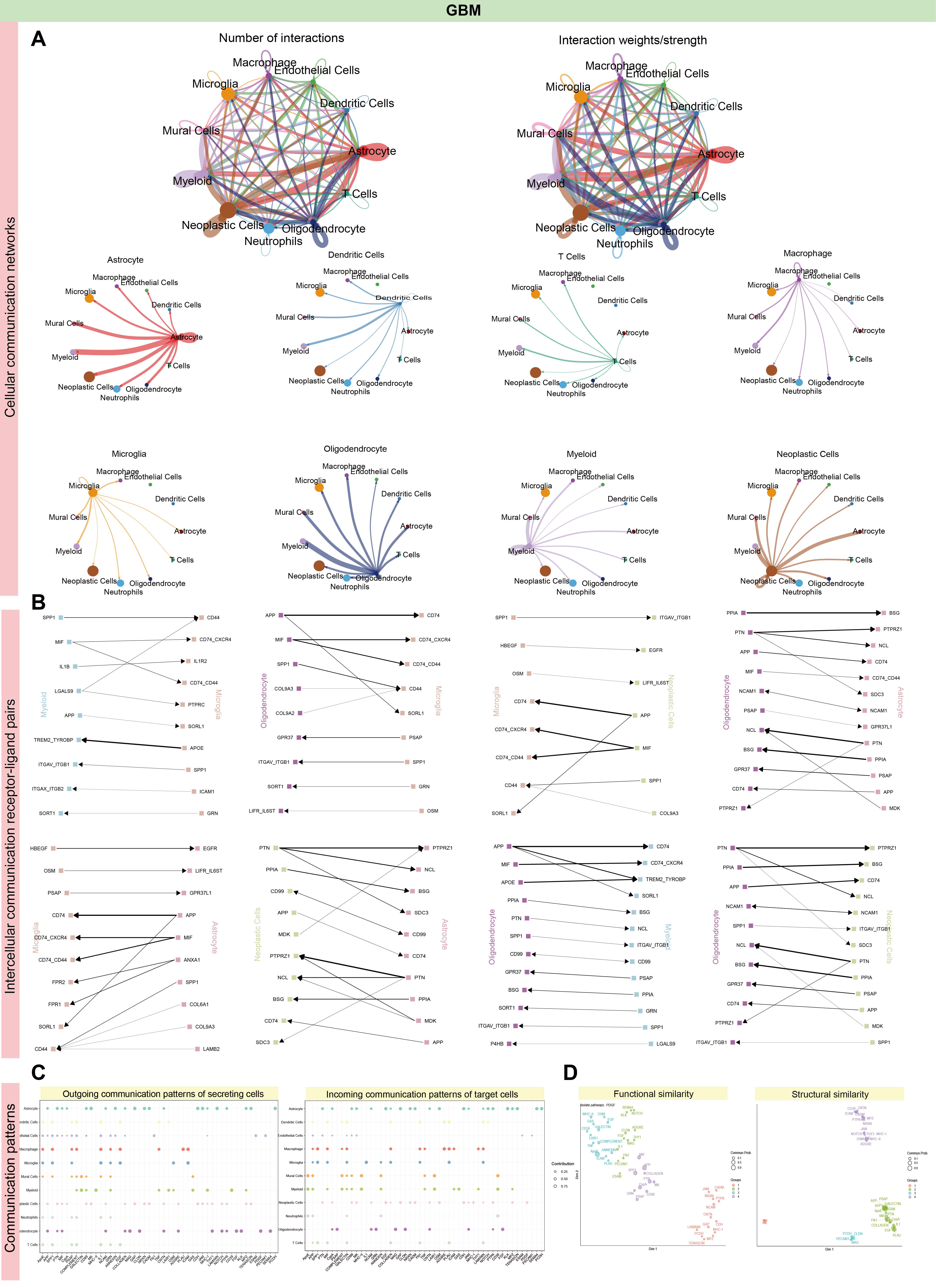


# Extended Data Fig.2 Extended data for cell communication analysis of GBM single-cell data.

**(A)** In GBM, intercellular interactions were particularly concentrated among specific cell types, with Astrocytes, Myeloid Cells, Neoplastic Cells, and Oligodendrocytes exhibiting the highest interaction counts. This dense network of interactions suggested a critical role for these cell types in the cellular communication within GBM. **(B-C)** A significant enrichment of the APOE or APP pathway was observed across most cell types, including Astrocytes, Oligodendrocytes, Microglia, Dendritic Cells, Macrophages, and T cells. The interactions involving APP-CD74 and APP-(TREM2+TYROBP) are especially prominent, indicating a key role for these pathways in cellular communication within GBM. The enrichment of the APP pathway in these cell types may contribute to the immune response and disease progression, highlighting the potential for targeted therapies. **(C)** The manifold and classification learning analysis of signaling networks provided a deeper understanding of the relationships between different signaling pathways. This analysis revealed how pathways may interact and contribute to the overall signaling network within GBM. **(D)** By quantifying the similarities between signaling pathways, we can identify groups of pathways that may share functional or structural roles within the cellular communication network. This approach helps to discern the complex interactions that drive disease progression in GBM. The distinct signaling patterns observed among immune cells, such as Microglia, Dendritic Cells, Macrophages, and T cells, suggest a coordinated response that could be targeted to enhance the immune response against GBM. The unique signaling patterns of Astrocytes and Oligodendrocytes, which were also enriched for the APP pathway, might offer specific targets for therapy to modulate disease progression. Understanding these signaling patterns was crucial for developing targeted therapies that could potentially disrupt disease-associated communication networks. The shared signaling patterns among immune cells may represent a common vulnerability that could be exploited to enhance the immune response against GBM, while the unique patterns of Astrocytes and Oligodendrocytes might offer avenues for selectively targeting these cells to modulate disease progression.


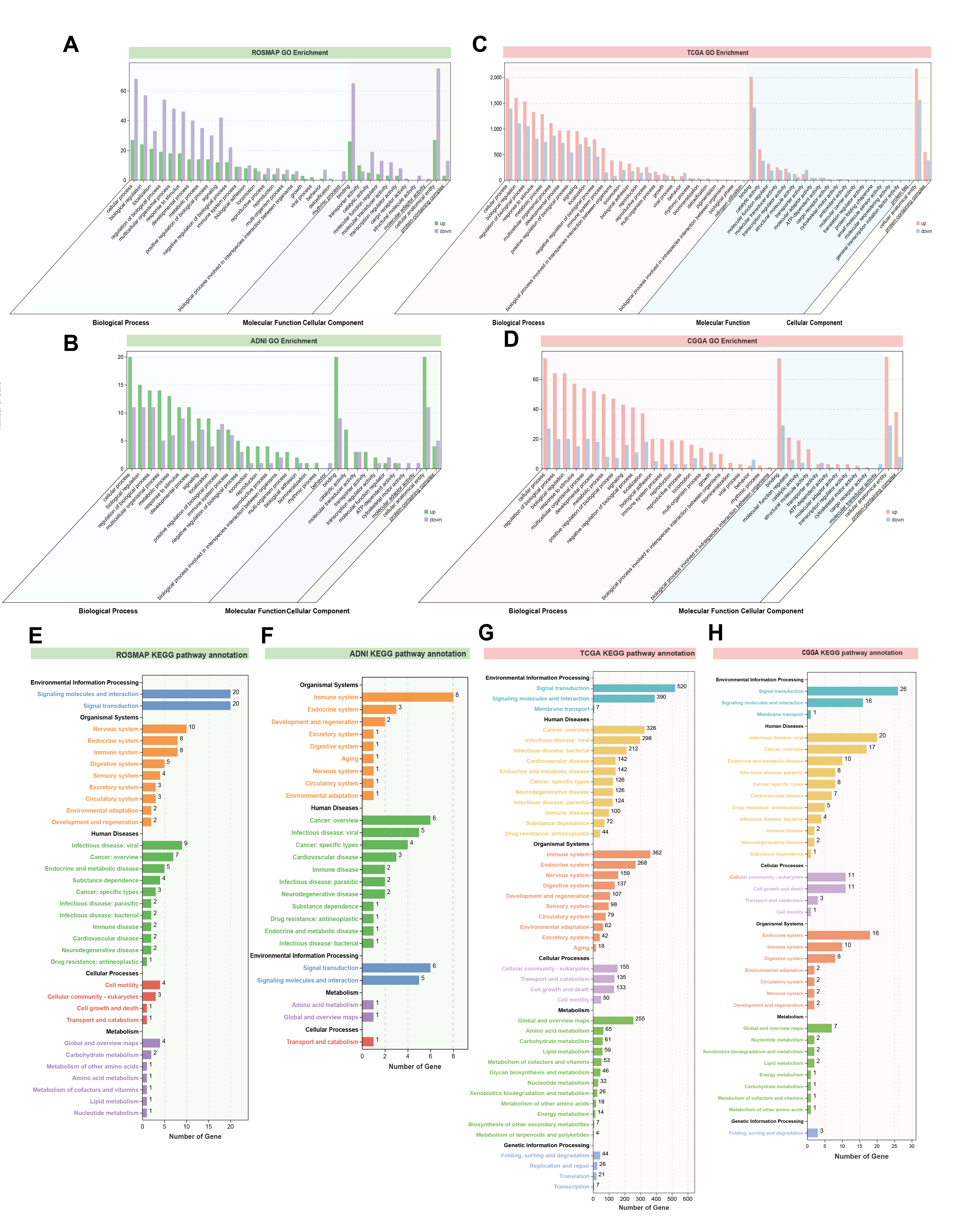


# Extended Data Fig.3 Extended data for transcriptomic characterization of AD and GBM.

The functional analysis of differentially expressed genes across ROSMAP, ADNI, TCGA, and CGGA databases has shed light on the molecular mechanisms underlying AD and GBM. **(A, E)** The ROSMAP dataset revealed that the differential genes were predominantly involved in nuclear chromosome segregation, suggesting a potential disruption in cellular processes related to the maintenance of genomic integrity, which could be a significant factor in the pathogenesis of AD. **(B, F)** In contrast, the ADNI dataset showed a primary association of differential genes with immune-related processes, particularly B cell activation and signaling pathways, indicating a crucial role for the immune system in the progression of AD. For GBM, **(C, G)** the TCGA dataset highlighted differential genes chiefly involved in protein localization and assembly, pointing towards potential alterations in cellular machinery that could contribute to tumor growth and maintenance. **(D, H)** The CGGA dataset revealed a significant implication of differential genes in chromosome segregation and mitotic processes, suggesting a role for these genes in the uncontrolled cell division characteristic of GBM. The distinct functional profiles of differentially expressed genes across these datasets underscore the heterogeneity in molecular pathways associated with AD and GBM. These insights are invaluable for understanding disease mechanisms and could guide the development of targeted therapies that address the specific molecular aberrations in each disease context.


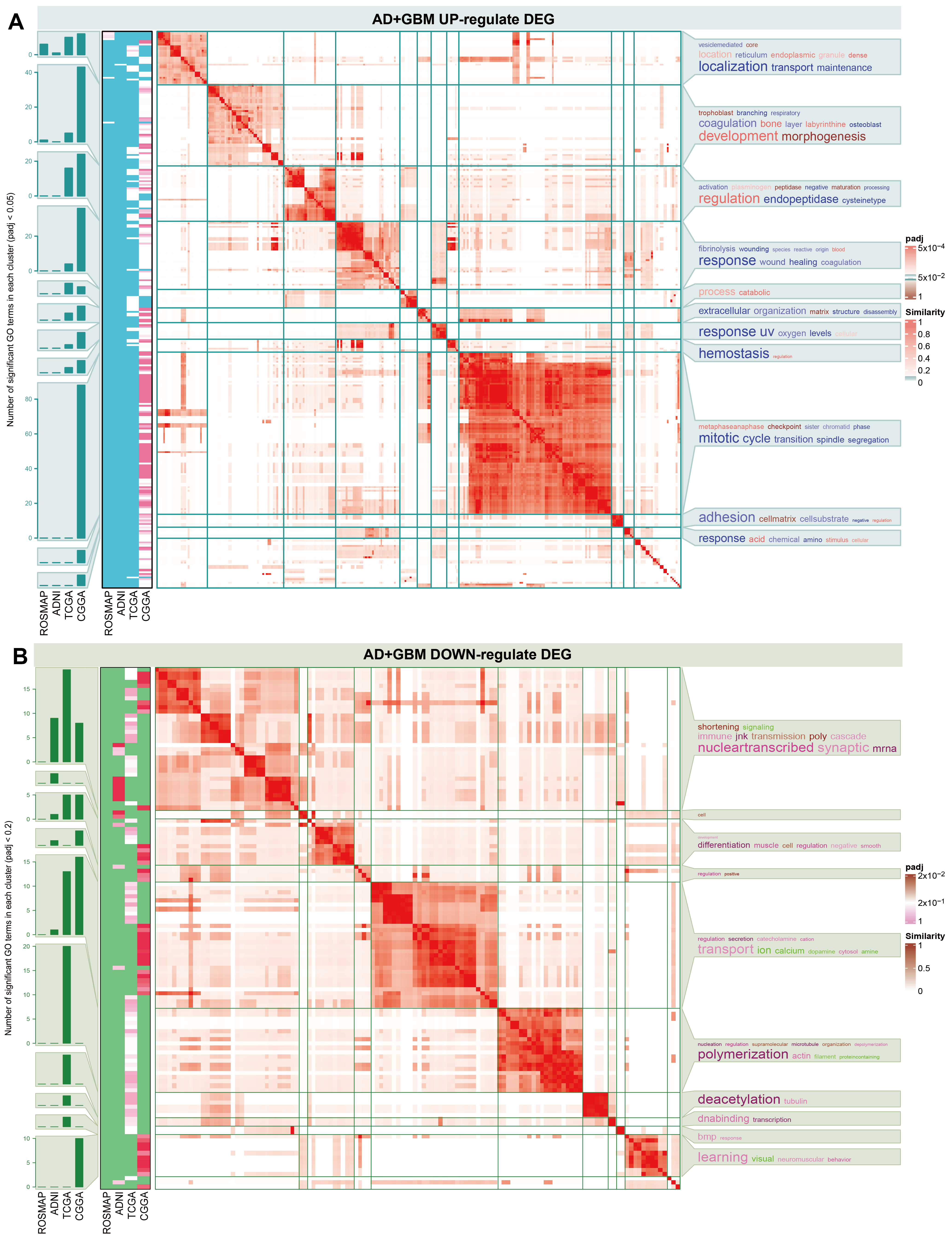


# Extended Data Fig.4 Overlapping patterns of gene enrichment observed in AD and GBM.

Consolidating the results from all differential expression analyses, the study revealed that the identified genes were most enriched in fundamental cellular processes, binding processes, and cellular anatomical entities. This comprehensive analysis provided a broad overview of the molecular changes associated with both AD and GBM. **(A) Up-Regulated Genes:** When categorizing the biological function terms, up-regulated genes were particularly enriched in processes such as mitosis, osteoblast morphogenesis, and protein-localized transcriptional maintenance. These enrichments indicate a potential role for these processes in the progression and characteristics of both AD and GBM. **(B) Down-Regulated Genes:** Conversely, down-regulated genes were most significantly enriched in nuclear transcription, synaptic signaling, and calcium ion transport. The downregulation of genes involved in these processes may contribute to the observed pathologies in AD and GBM, affecting critical cellular functions and intercellular communications. The distinct yet overlapping patterns of gene enrichment observed in both diseases highlight the complexity of their molecular landscapes. Understanding these patterns is crucial for developing targeted therapies and could provide insights into the shared and disease-specific mechanisms that drive AD and GBM.


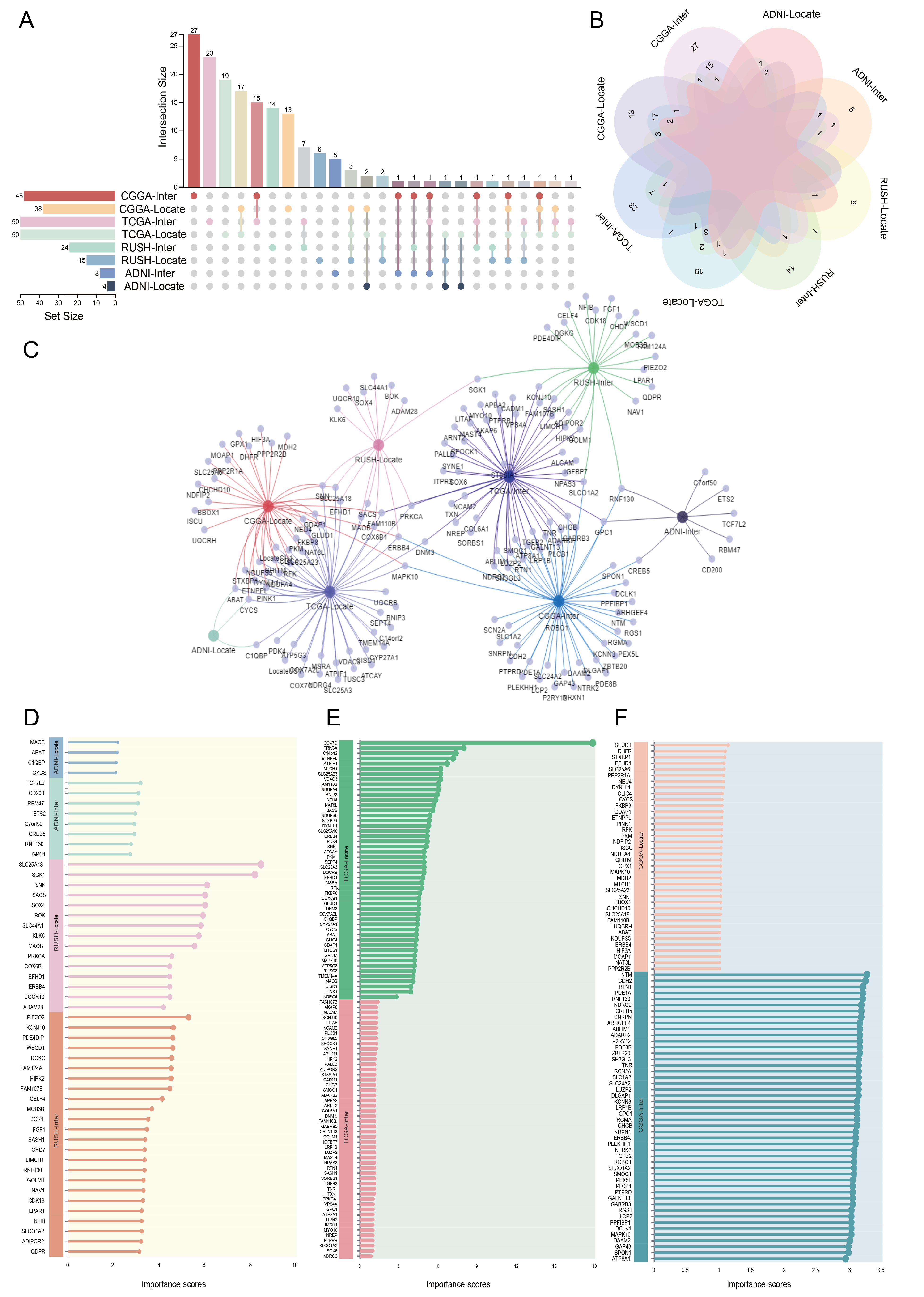


# Extended Data Fig.5 Integrative analysis reveals key mitochondrial markers and their intersections in AD and GBM.

**(A-C)** By integrating the variable importance from the top three predictive models, we systematically identified key markers within the mitochondrial epistasis (MT-Inter) and mitochondrial localization (MT-Locate) categories across four databases: ADNI, ROSMAP, TCGA, and CGGA. For ADNI, 9 MT-Inter and 5 MT-Locate markers were identified, ROSMAP yielded 25 MT-Inter and 16 MT-Locate markers, and CGGA revealed 39 MT-Inter and 49 MT-Locate markers. In the TCGA dataset, the top 50 marker genes were selected for further analysis. The network diagram analysis revealed significant intersections among these gene sets, with the TCGA MT and CGGA MT showing the highest number of gene intersections (17). Notably, the gene *ERBB4* emerged as a significant contributor across multiple models, including RUSH- Locate, CGGA-Locate, TCGA-Locate, and CGGA-Inter. **(D-F)** Consolidating all biomarkers with high importance, we curated a list of 24 candidate cell-specific mitochondria-associated markers that demonstrated significant contributions in at least one dataset for both AD and GBM. Among these, 16 were mitochondrial epistasis genes, and 13 were mitochondrial localization genes. Notably, five genes—*ERBB4, ABAT, FAM110B, MAPK10,* and *PRKCA*—were classified as both mitochondrial epistasis and mitochondrial localization genes.





# Extended Data Fig.6 The specific expression patterns of the identified candidate marker genes in various cell types of AD.

This figure illustrated the expression patterns of the identified candidate marker genes in various cell types affected by AD. The genes were predominantly expressed in Oligodendrocytes and exhibited distinct distribution differences across neural cells. Notably, significant disparities in gene expression were observed between astrocytes and oligodendrocytes, which may have reflected the heterogeneity in cellular roles and responses within the disease context. The unique expression profiles provided insights into the molecular mechanisms of AD and highlighted the potential of these markers for targeted therapeutic development.





# Extended Data Fig.7 The specific expression patterns of the identified candidate marker genes in various cell types of GBM.

This figure detailed the expression patterns of the candidate marker genes in different cell types within the context of GBM. These genes showed a strong predilection for expression in Neoplastic cells, with notable differences in expression levels when compared to immune cells. Similar to Alzheimer's Disease (AD), there was a pronounced distinction in gene expression between Astrocytes and Oligodendrocytes.

Understanding these expression patterns was crucial for deciphering the cellular interactions in GBM and could have guided the creation of cell-specific treatments that exploited the mitochondrial-associated characteristics of these cells.


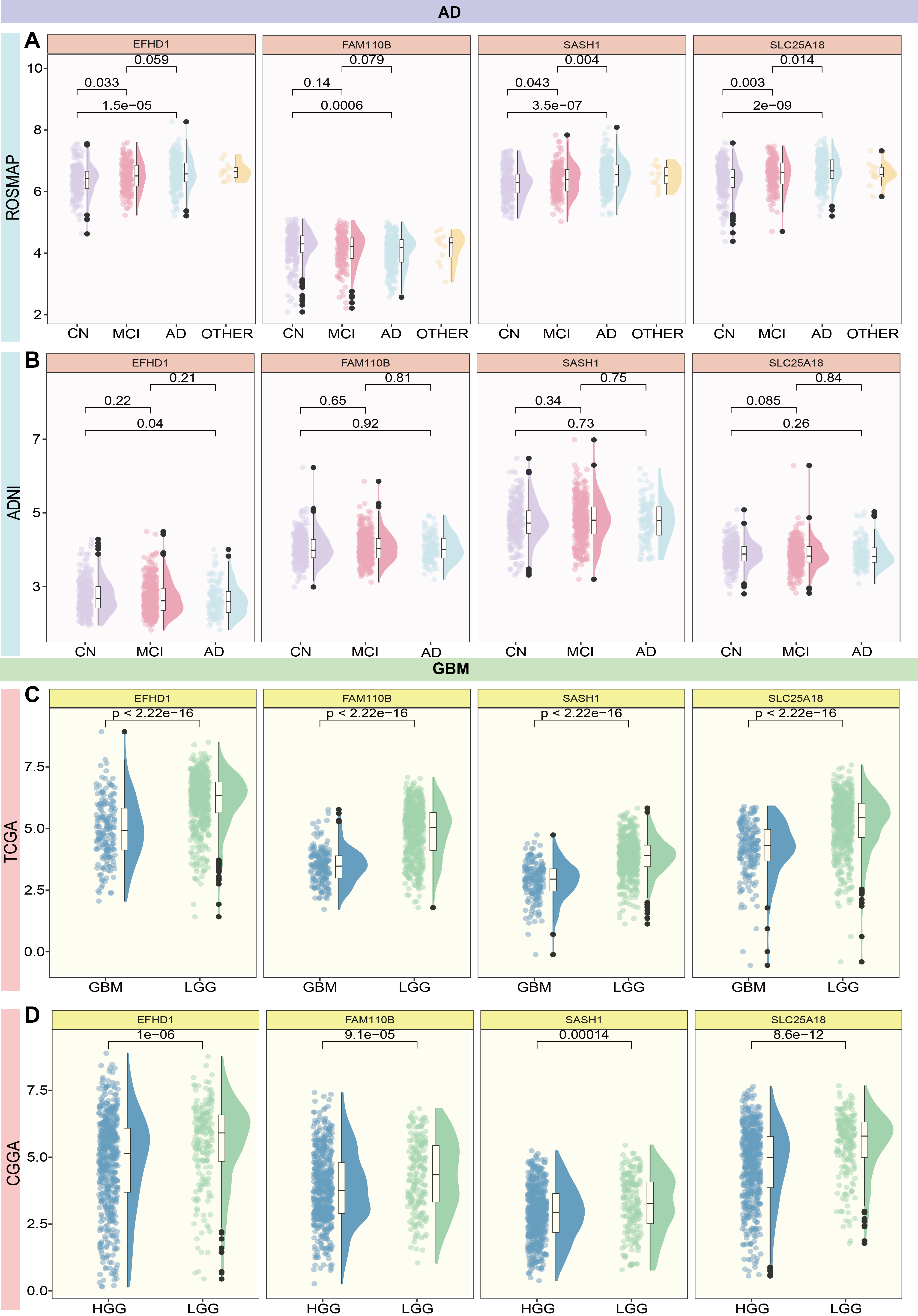


# Extended Data Fig.8 Expression patterns for four candidate genes in four independent datasets.

We performed an additional screening of integrated gene expression data, identifying four genes that exhibit differential expression in both AD and GBM. These genes, *EFHD1, SASH1, FAM110B, and SLC25A18*, were found to have significant expression patterns that differ between the two diseases, highlighting their potential role in disease pathogenesis. The identification of these genes through a comprehensive analysis of gene expression profiles underscored the importance of a multi-omics approach in understanding the molecular underpinnings of complex neurological disorders. The differential expression of *EFHD1, SASH1, FAM110B,* and *SLC25A18* in both AD and GBM suggested that these genes may contribute to disease mechanisms in distinct but potentially interconnected ways. Further investigation into the functions of these genes and their interactions within the cellular context could have provided valuable insights into the pathophysiology of AD and GBM. This knowledge may have facilitated the development of targeted therapies and personalized medicine strategies, ultimately improving clinical outcomes for patients affected by these diseases.


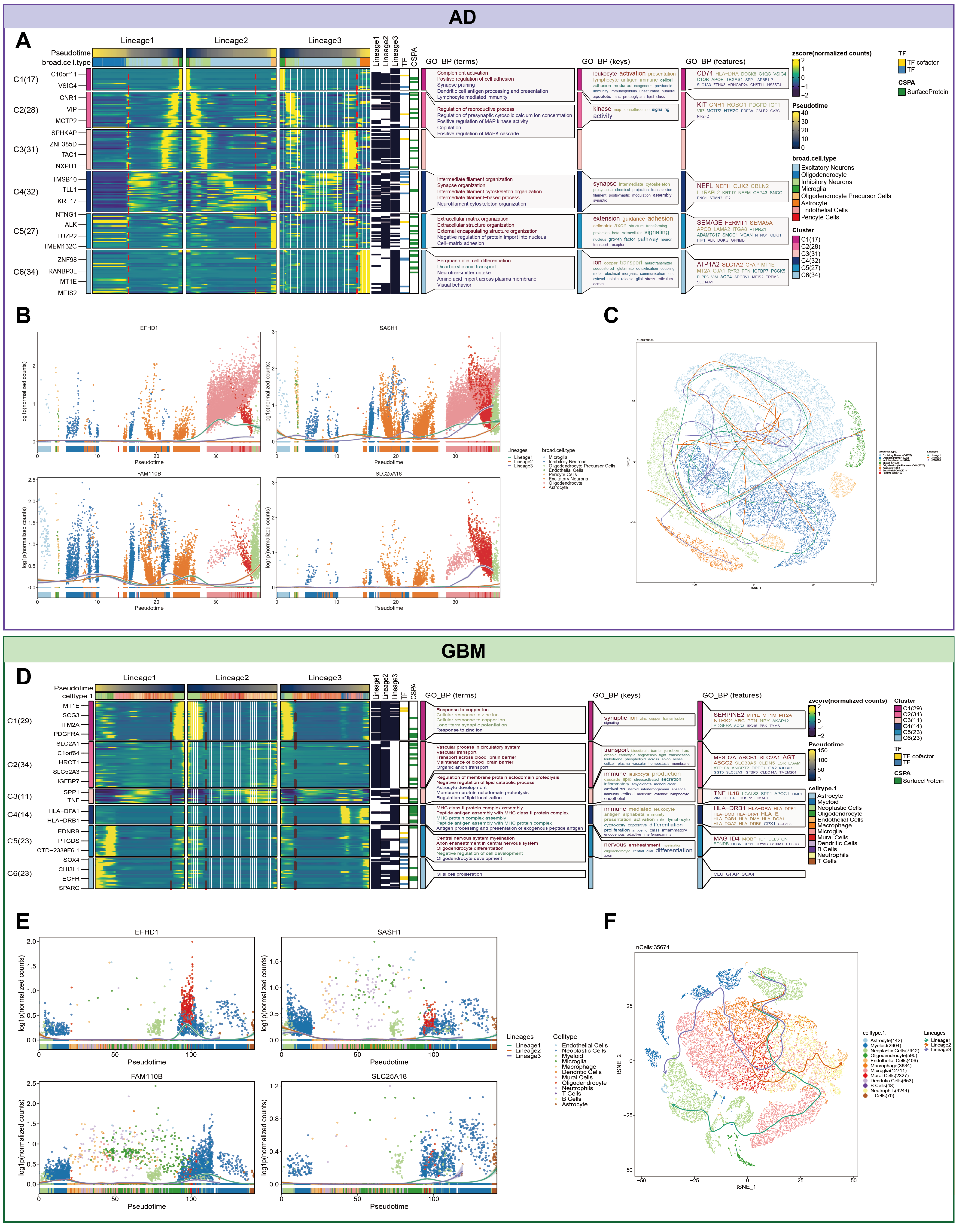


# Extended Data Fig.9 Lineage-specific gene expression dynamics in AD and GBM.

The dynamic features within AD and GBM sn/scRNA-seq datasets were presented, highlighting the complexity and diversity of cellular responses in these diseases. The heatmap presented illustrates three distinct lineages and six feature clusters per dataset, each characterized by unique biological functions.

1. **In AD:**

- *Cluster 1 (C1)* was enriched for immune processes, such as complement activation and cell adhesion, indicating a significant role in the immune response within the disease context.
- *Cluster 2 (C2)* was involved in the regulation of the MAPK cascade, a key signaling pathway in cellular communication.
- *Cluster 4 (C4)* pertained to intermediate filaments and the cytoskeleton, reflecting the involvement of structural components in disease progression.

**(D) In GBM:**

- *Cluster 1 (C1)* showed a response to essential metal ions such as copper and zinc, and is associated with synaptic potentiation, pointing to the importance of metal homeostasis in tumor biology.
- *Cluster 2 (C2)* correlated with the blood-brain barrier, suggesting a role in the tumor's interaction with the brain's protective barrier.
- *Cluster 3 (C3)* was linked to membrane proteins and lipolytic processes, indicating a potential involvement in cellular transport and metabolism.

**(B-C)** The tSNE downscaling maps provided further insights into the potential evolutionary paths of various cell types in AD and GBM. In AD, the expression levels of astrocytes, oligodendrocytes, and oligodendrocyte precursor cells were notably higher in the later stages across the three spectral time series. Specifically, *EFHD1* exhibits increased expression at the late pseudotime stage of Lineage 1, while *SASH1* showed elevated expression at the late pseudotime stages across Lineages 1, 2, and 3. The expression of *FAM110B* oscillated similarly across different lineages and time periods, and *SLC25A18* displayed increased expression at the late pseudotime stage of Lineage 3.

**(E-F)** In GBM, *EFHD1* expression was heightened in oligodendrocytes and tumor cells during the middle and late pseudotime stages of Lineage 1. *SASH1* demonstrated a decreasing expression trend from pre-pseudotime tumor cells and myeloid cells. *FAM110B* expression was upregulated in tumor cells and some oligodendrocytes during the middle and late stages of Lineages 1 and 3. *SLC25A18* expression was elevated in tumor cells and a subset of astrocytes and oligodendrocytes at the late pseudotime stages of Lineages 1 and 3. These findings underscored the dynamic and lineage-specific gene expression patterns in AD and GBM, offering valuable insights into the molecular mechanisms underlying these diseases and potential avenues for therapeutic intervention.


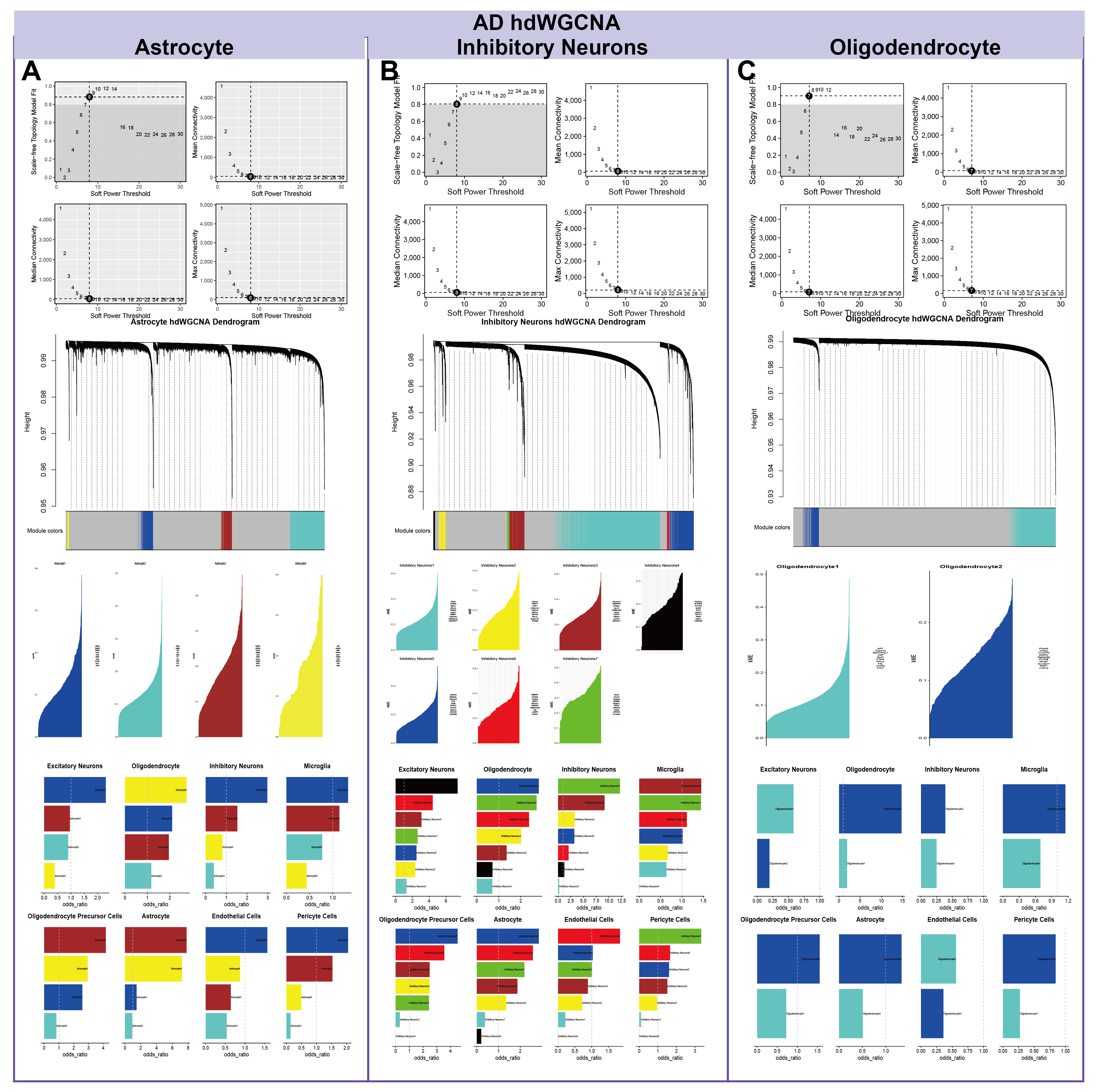


# Extended Data Fig.10 Construction of hdWGCNA network for AD.

The process of establishing gene co-expression networks at the single-cell level to explore the interaction patterns among candidate genes, a critical step in understanding the molecular landscape of cellular heterogeneity in complex diseases. The analysis began with a quality-controlled and downscaled Seurat object, which served as the input for hdWGCNA. This object was essential for accurate clustering and network construction. The meta units were then constructed, and the appropriate soft threshold power was determined through a parameter sweep using the function TestSoftPowers. The results of this parameter sweep were visualized with the PlotSoftPowers function, providing a clear indication of the optimal soft threshold for network construction. A sample clustering tree diagram and cluster analysis of all samples were presented, demonstrating the absence of significant outliers in the sample. This ensures the reliability of the subsequent module identification and gene clustering. **(A)** Astrocytes were categorized into four distinct modules, suggesting diverse roles and gene expression profiles within this cell type. **(B)** Inhibitory neurons were further differentiated into seven modules, highlighting the complexity of their regulatory networks and potential functional specialization. **(C)** Oligodendrocytes were divided into two modules, indicating a more streamlined yet still critical set of gene expression patterns.


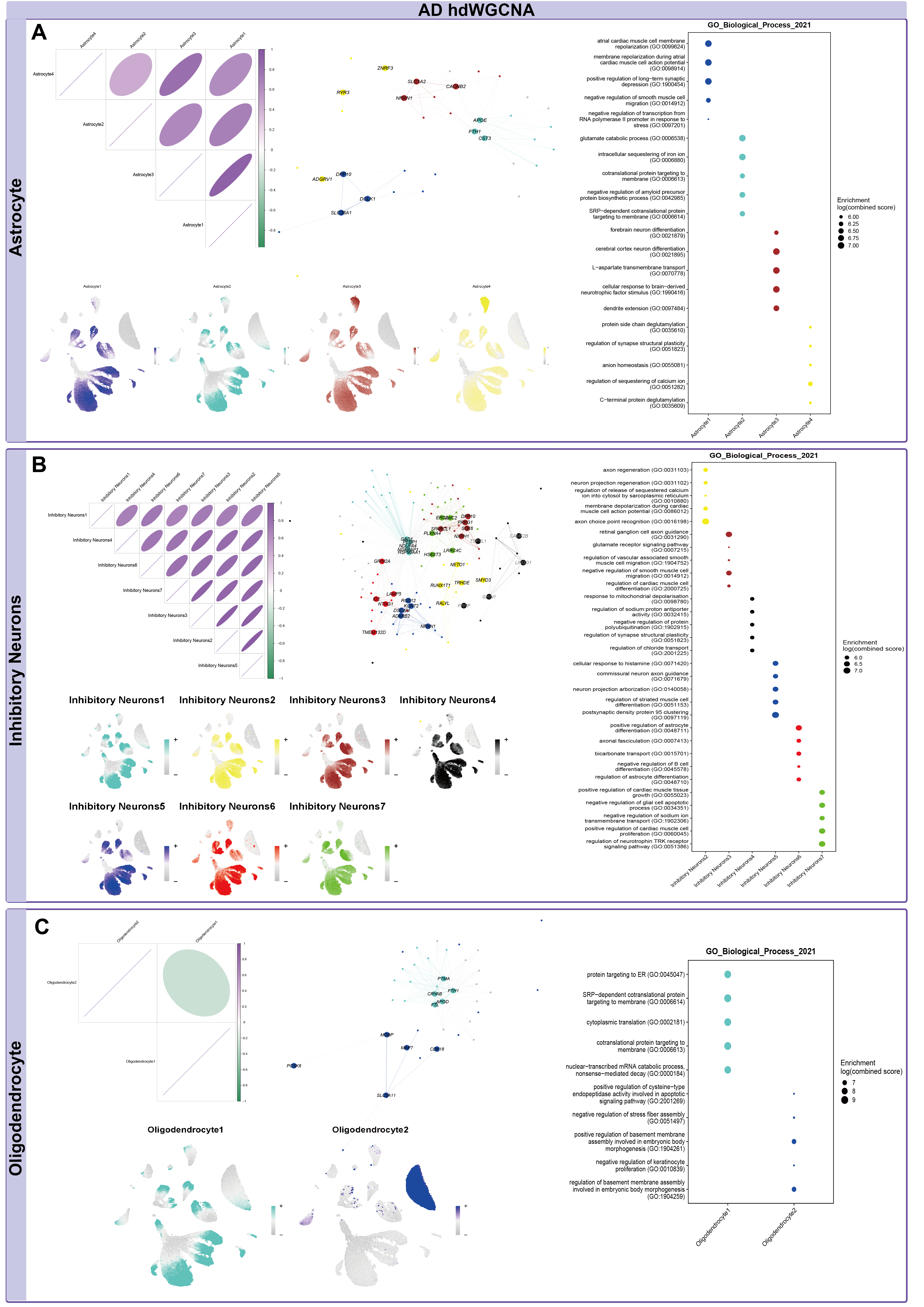


# Extended Data Fig.11 Detailed analysis of the co-expression modules of key cell types in AD.

The gene enrichment analysis has unveiled specific associations between candidate genes and cell types, providing insights into the molecular functions and pathways active within distinct cellular modules in the context of neurological diseases.

- *SASH1* was found to be associated with Astrocytes3, Inhibitory Neurons3, and Oligodendrocytes2, suggesting its role in diverse cellular processes across different cell types.
- *FAM110B* showed associations with Astrocytes and Inhibitory Neurons3, indicating a potential involvement in neuronal function and astrocyte activity.
- EFHD1 was specifically associated with oligodendrocytes1, pointing to a possible specialization in this cell type.

Furthermore, **(A)** the Astrocyte2 module demonstrated the strongest association with Excitatory Neurons and was functionally enriched for the glutamate catabolic process (GO:0006538) and intracellular sequestration of iron ions (GO:0006880). These enrichments indicate a role in neurotransmitter metabolism and iron homeostasis, which are critical for neuronal health. Synergistic gene expression between excitatory and inhibitory neurons was observed, highlighting the coordinated interaction between these cell types in regulating neural activity. **(B)** The Inhibitory Neurons3 module was particularly involved in retinal ganglion cell axon guidance (GO:0031290) and the glutamate receptor signaling pathway (GO:0007215), emphasizing the importance of inhibitory signals in neural circuit development and function. **(C)** The Oligodendrocyte1 and Oligodendrocyte2 modules were enriched for endoplasmic reticulum proteins (GO:0045047) and positive regulation of basement membrane assembly in embryoid body morphogenesis (GO:1904261), respectively. These enrichments suggest a role in protein processing and the development of the extracellular matrix, which are essential for oligodendrocyte function and central nervous system integrity.


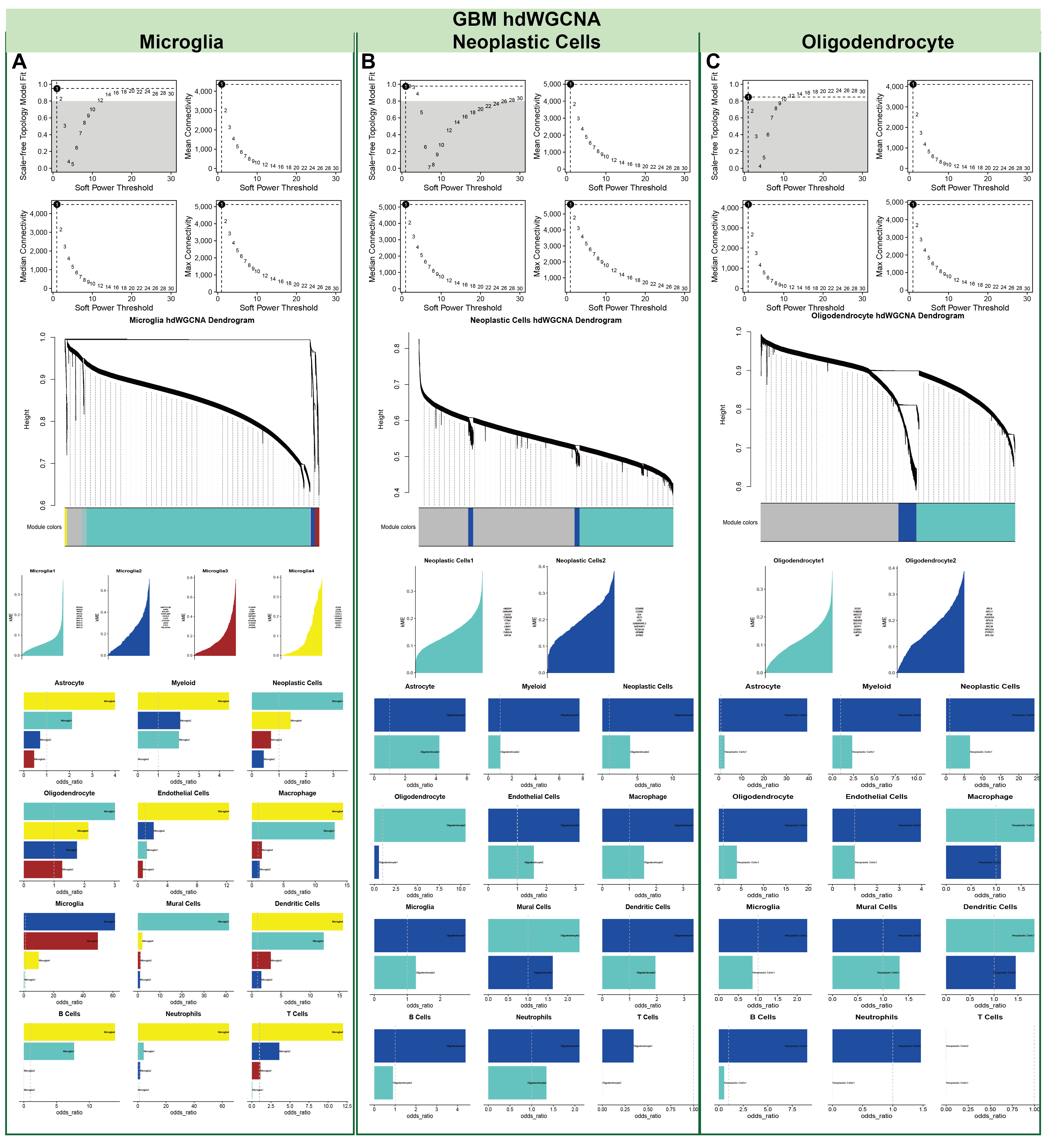


# Extended Data Fig.12 Construction of hdWGCNA network for GBM.

In the context of GBM, the gene co-expression network analysis has delineated distinct cellular modules, revealing the complex molecular landscape of this aggressive brain tumor. **(A)** The microglia, the resident immune cells of the central nervous system, were categorized into four distinct modules, indicative of their diverse roles and activation states within the tumor microenvironment. **(B)** Neoplastic Cells, which are the cancerous cells of GBM, were divided into two modules, suggesting the existence of different subtypes or stages within the tumor population. This division may reflect the heterogeneity of the tumor cells and their varying capacities for proliferation, invasion, and response to therapy. **(C)** Oligodendrocytes, the cells responsible for producing myelin in the brain, were also divided into two modules. This distinction may represent differences in the cells' responses to the tumor environment, their involvement in tumor-associated processes, or their potential as targets for tumor-induced changes. The division of these cell types into modules provides a foundation for further investigation into the specific gene expression patterns and functional roles within GBM. Understanding the molecular signatures of each module can offer insights into GBM pathophysiology and identify potential therapeutic targets that may help in developing more effective treatment strategies for this devastating disease.


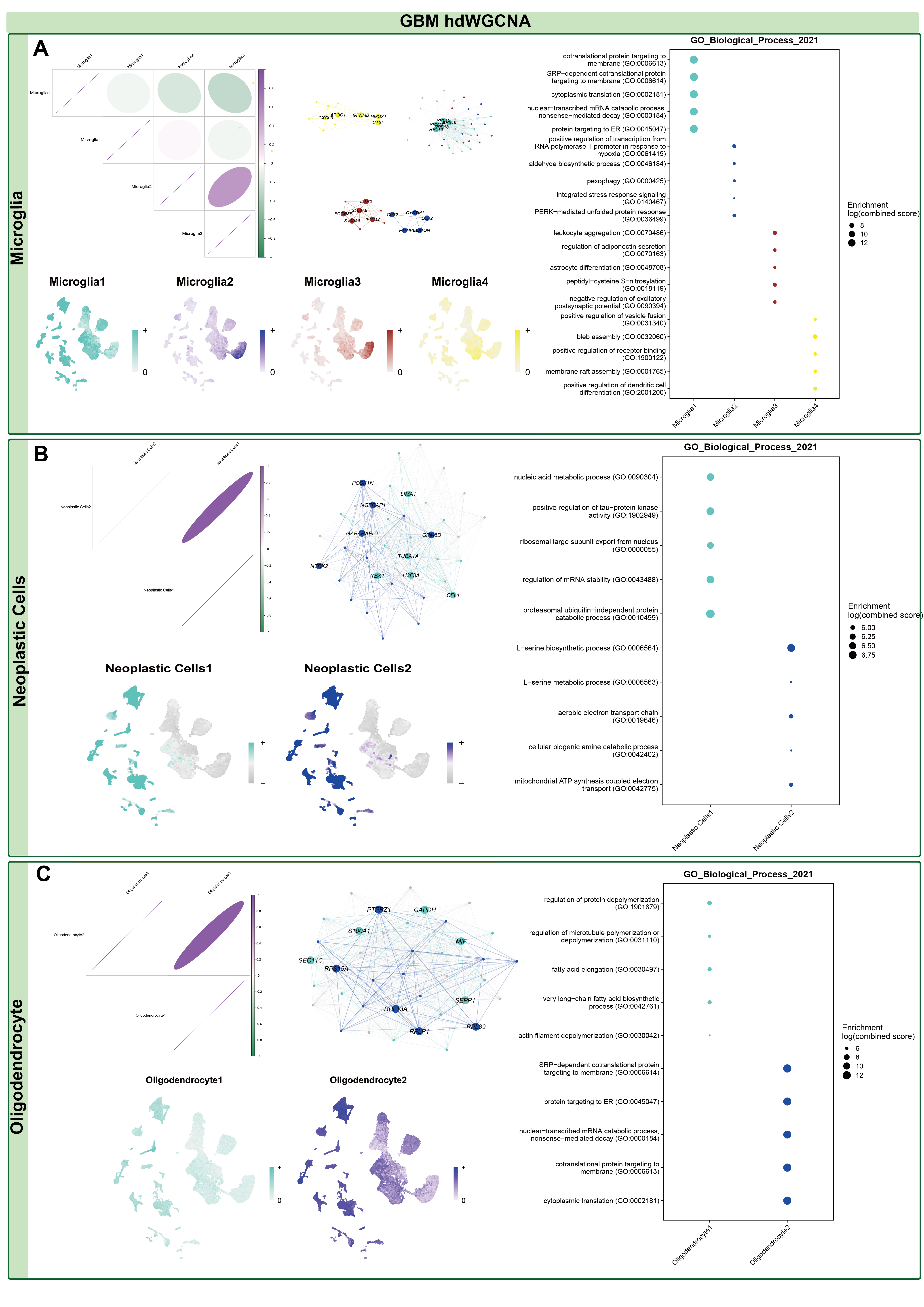


# Extended Data Fig.13 Detailed analysis of the co-expression modules of key cell types in GBM.

The gene enrichment analysis in GBM has identified specific associations between candidate genes and cellular modules, offering insights into the molecular mechanisms at play within the tumor microenvironment.

- *SASH1* was enriched in Microglia3 and Oligodendrocytes1, suggesting its role in immune response and oligodendrocyte function.
- *FAM110B* was associated with Neoplastic Cells1 and Oligodendrocytes2, indicating its potential involvement in tumor cell biology and oligodendrocyte-related processes.

**(A)** Microglia3, one of the modules of microglia, was found to be involved in several biological processes:

- Leukocyte aggregation (GO:0070486), which is critical for immune cell interactions within the tumor.
- Adiponectin regulation (GO:0070163), implicating a role in metabolic regulation within the tumor microenvironment.
- Astrocyte differentiation (GO:0048708), highlighting a potential influence on the development of other glial cells.
- Excitatory postsynaptic potential (GO:0090394), indicating a connection to synaptic transmission, which may be affected by microglial activity.

**(C)** Oligodendrocyte1 and Oligodendrocyte2 were linked to distinct biological functions:

- The regulation of protein depolymerization (GO:1901879) in Oligodendrocyte1, which may relate to the dynamics of cytoskeletal elements important for myelin formation and maintenance.
- Protein targeting to the endoplasmic reticulum (GO:0045047) in Oligodendrocyte2, reflecting a role in protein processing and quality control, essential for cellular function and survival.

These enrichments provide a deeper understanding of the cellular roles and interactions in GBM, with implications for disease mechanisms and potential therapeutic targets. The specific biological processes highlighted by the gene enrichment analysis suggest pathways that may be targeted to modulate the tumor microenvironment and impact GBM progression.





# Extended Data Fig.14 Major pathways involving *EFHD1, SASH1, FAM110B*, and *SLC25A18.*

(A) Transcriptional Regulation by the AP-2 (TFAP2) Family of Transcription Factors (R-HSA-8864260.2)

- Key Interaction: *EFHD1* and *ESR1* interaction (score = 0.527).
- Mechanism: *EFHD1* potentially influences calcium-dependent transcriptional co-activators or repressors that modulate *ESR1* activity. *TFAP2* and *ESR1* co-occupy promoter regions, integrating signals from *EFHD1*-mediated mitochondrial processes to regulate TFAP2-dependent genes.
- Functional Significance: This interaction integrates transcriptional control of hormone-related genes, supported by cofactors like ATAD2, and contributes to cellular growth and angiogenesis through VEGFA regulation.

(B) TP53 Regulates Transcription of Cell Cycle Genes (R-HSA-6791312.5)

- Key Interaction: *SASH1* and *SFN* interaction (score = 0.536).
- Mechanism: *SFN* (14-3-3σ), a crucial effector of *TP53*, enforces G2/M checkpoint arrest by stabilizing CDK inhibitors and preventing mitotic entry. *SASH1* enhances this process by interacting with *SFN*, amplifying *TP53*-mediated responses to DNA damage and cellular stress.
- Functional Significance: This interaction contributes to tumor suppression by coordinating cell cycle arrest, DNA repair, and apoptosis pathways, crucial for genomic stability.

(C) Deregulated CDK5 Triggers Multiple Neurodegenerative Pathways in Alzheimer's Disease (R-HSA-8862803.4)

- Key Interaction: *FAM110B* and *YWHAE (14-3-3ε)* interaction (score = 0.564).
- Mechanism: Deregulated *CDK5* activates neurodegenerative processes such as Tau (MAPT) hyperphosphorylation, mitochondrial dysfunction, and oxidative stress. The *FAM110B-YWHAE* interaction stabilizes the cytoskeleton and modulates stress responses. *YWHAE* regulates apoptotic proteins like *BCL2L1*, mitochondrial antioxidants (*SOD2*), and transcription factors (*FOXO3*), supporting neuronal survival.
- Functional Significance: Together, *FAM110B* and *YWHAE* mitigate CDK5-induced damage, regulating Tau aggregation, oxidative stress, and neuronal apoptosis, making them potential therapeutic targets in AD.

(D) Malate-Aspartate Shuttle (R-HSA-9856872.1)

- Key Role: *SLC25A18* as a mitochondrial carrier protein.
- Mechanism: *SLC25A18* transports L-glutamate (L-Glu) and protons (H⁺) across the mitochondrial inner membrane. It links glutamate metabolism to the TCA cycle by importing glutamate into the mitochondrial matrix, where it is converted into 2-oxoglutarate, a critical TCA cycle intermediate.
- Functional Significance: This process supports energy production, biosynthesis (e.g., aspartate and nucleotide synthesis), and mitochondrial homeostasis. By utilizing the mitochondrial proton gradient, *SLC25A18* integrates metabolite transport with oxidative phosphorylation, ensuring metabolic flexibility and cellular energy balance.


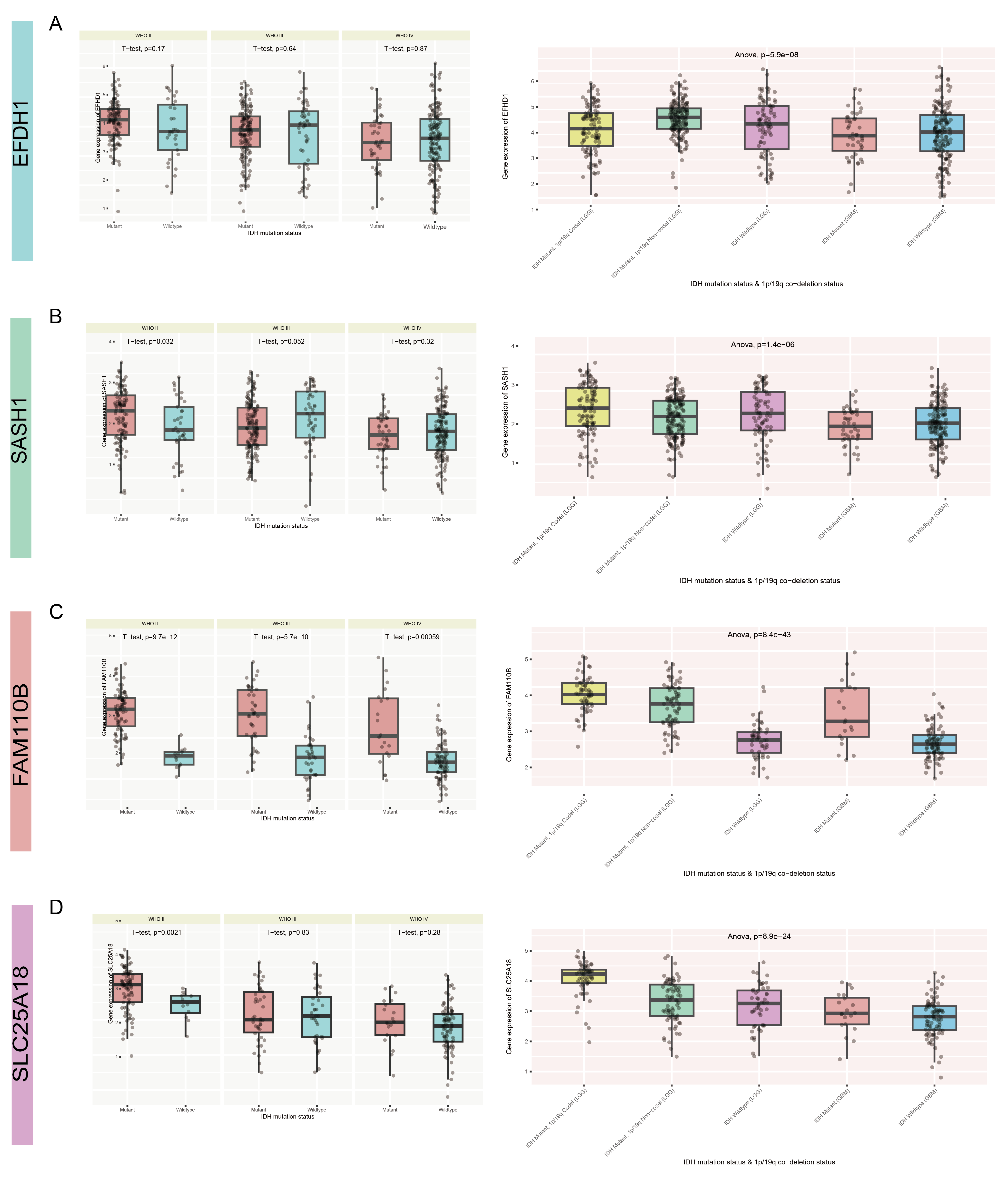


# Extended Data Fig.15 Differential expression of four key marker genes across IDH subtypes and Glioma severity grades*.*

The expression patterns of four key marker genes (A: *EFHD1*, B: *SASH1*, C: *FAM110B*, D: *SLC25A18*) across different IDH subtypes and glioma severity grades based on the CGGA dataset. The left panels show the expression levels stratified by IDH mutation status (wild-type or mutant) and WHO grades (II, III, and IV). The right panels illustrate the expression differences among IDH subtypes, including IDH wild-type, IDH mutant without 1p/19q codeletion, and IDH mutant with 1p/19q codeletion (LGG). FAM110B exhibited the most significant differential expression, with consistently high expression in IDH mutant gliomas across all severity grades. SLC25A18 showed the highest expression in IDH mutant gliomas of WHO grade II and in low-grade gliomas (LGG) with 1p/19q codeletion. These findings underscore the potential of these genes as biomarkers for glioma classification and severity assessment.
